# *ACKR1*/Duffy‐null genotype testing for clozapine: A guideline developed by the UK Centre of Excellence in Regulatory Science and Innovation in Pharmacogenomics (CERSI‐PGx)

**DOI:** 10.1002/bcp.70576

**Published:** 2026-05-13

**Authors:** Stephen Murtough, Oriella Stellakis, Daisy Mills, Blanca Bjourson, Vicky Chaplin, Dharmisha Chauhan, Bev Chipp, Marius Cotic, Jana de Villiers, Olubanké Dzahini, Frances Elmslie, Katie Evans, Shreyans Gandhi, Dyfrig A. Hughes, Huajie Jin, Daniele Panconesi, Anna Skowronska, Sanjay M. Sisodiya, Ed Silva, Vicky Stinton, Sara Stuart‐Smith, Sarah Tarrant, David Taylor, Lauren Varney, James T. R. Walters, Michelle Wood, Jessica Woodley, Cinzia Dello Russo, Munir Pirmohamed, Elvira Bramon

**Affiliations:** ^1^ Division of Psychiatry University College London London UK; ^2^ Belfast Health and Social Care Trust Belfast UK; ^3^ Genomics Unit National Health Service England London UK; ^4^ North Thames Genomic Medicine Service Alliance London UK; ^5^ The Side‐By‐Side Network London UK; ^6^ The Royal College of Psychiatrists London UK; ^7^ High Secure Intellectual Disability Service for Scotland and Northern Ireland Carstairs UK; ^8^ Pharmacy Department South London and Maudsley NHS Foundation Trust London UK; ^9^ Institute of Pharmaceutical Science King's College London London UK; ^10^ South East Genomic Medicine Service Alliance London UK; ^11^ Cardiff and Vale University Health Board Cardiff UK; ^12^ Department of Haematology King's College Hospital NHS Foundation Trust London UK; ^13^ Centre for Health Economics and Medicines Evaluation, North Wales Medical School Bangor University Bangor UK; ^14^ King's Health Economics, Institute of Psychiatry, Psychology and Neuroscience King's College London London UK; ^15^ Birmingham Women's and Children's NHS Foundation Trust Birmingham UK; ^16^ Research Department of Epilepsy, Queen Square Institute of Neurology University College London London UK; ^17^ Specialist Community Forensic Team for People With Learning Disability and Autism Hollins Park Hospital Warrington UK; ^18^ North West Genomic Laboratory Hub Manchester University NHS Foundation Trust Manchester UK; ^19^ NHS Blood and Transplant Bristol UK; ^20^ Centre for Neuropsychiatric Genetics and Genomics, Division of Psychological Medicine and Clinical Neurosciences Cardiff University Cardiff UK; ^21^ All Wales Medical Genomics Service Cardiff UK; ^22^ Department of Pharmacology and Therapeutics, Institute of Systems, Molecular and Integrative Biology University of Liverpool Liverpool UK; ^23^ Department of Translational Medicine and Surgery, Section of Pharmacology Università Cattolica del Sacro Cuore – Fondazione Policlinico Universitario A. Gemelli, IRCCS Rome Italy; ^24^ The Wolfson Centre for Personalised Medicine, Centre for Drug Safety Science University of Liverpool Liverpool UK; ^25^ North London NHS Foundation Trust London UK

**Keywords:** *ACKR1*, *ACKR1*/*DARC*‐associated neutropenia, clozapine, Duffy‐null associated neutrophil count, Parkinson's disease, pharmacogenomics, treatment‐resistant schizophrenia

## Abstract

Clozapine is licenced for treatment‐resistant schizophrenia and psychosis in Parkinson's disease. In the United Kingdom, there is a mandatory requirement for absolute neutrophil count (ANC) and white blood cell count (WBC) monitoring to safeguard against agranulocytosis. Some people have naturally low ANCs without increased infection risk, caused by a homozygous T > C variant in *ACKR1*, commonly called the Duffy‐null genotype. This condition is known as ADAN (*ACKR1*/*DARC*‐associated neutropenia) and synonyms include DANC (Duffy‐null associated neutrophil count) and BEN (benign ethnic neutropenia). It is usual UK practice to lower WBC/ANC thresholds for people confirmed to have ADAN. However, ADAN often remains undetected, resulting in unnecessary discontinuation and exclusion from clozapine. This CERSI‐PGx guideline provides a framework to offer *ACKR1* genotype testing within the existing clinical pathway. We recommend three eligibility criteria, including pre‐emptive testing for all people starting clozapine, testing for people registered in the Central Non‐Rechallenge Database and reactive testing following a below‐threshold blood result (defined as ‘amber’ or ‘red’). We recommend that people with the Duffy‐null genotype should be monitored using revised WBC/ANC thresholds for ADAN. Regardless of *ACKR1* genotype, haematology input is required for people returning a WBC < 2.0 × 10^9^/L and/or ANC < 1.0 × 10^9^/L who present with a key clinical feature, such as sustained temperature ≥38°C. Finally, we summarize health economic evidence, estimating in the first year of testing, 129 people with the Duffy‐null genotype could be identified, resulting in savings ranging from £42 732 to £727 990. We propose *ACKR1* testing as a cost‐effective approach for facilitating access to clozapine, the optimal therapy for treatment‐resistant schizophrenia.

## 1 BACKGROUND AND OVERVIEW

Clozapine is the only licenced medicinal product for treatment‐resistant schizophrenia.^1^, ^2^, ^3^, ^4^, ^5^, ^6^ This refers to people who have not responded to two or more antipsychotic drugs (including at least one second‐generation antipsychotic).^1^, ^4^, ^7^ Approximately 30% of people with schizophrenia are treatment resistant,^8^ and they have an increased risk of hospitalization and suicide.^9^ When compared with other antipsychotic drugs, clozapine is the most effective therapy for treatment‐resistant schizophrenia,^10^ with a response rate of 50–60%.^11^ Additionally, clozapine is licenced for psychosis in Parkinson's disease (see Section 2),^1^, ^4^, ^5^, ^6^ although it is underused for this indication due to requirements for blood monitoring and few established clozapine services in neurology settings.^12^, ^13^

### 1.1 The UK monitoring system for clozapine

For some people, clozapine can cause serious adverse drug reactions, including neutropenia (absolute neutrophil count [ANC] < 1.5 × 10^9^/L) and agranulocytosis (ANC < 0.5 × 10^9^/L),^1^, ^2^ with prevalence rates of 3.8% and 0.4%, respectively.^14^ Agranulocytosis can be fatal due to the high risk of infection.^15^ Consequently, most countries recommend or mandate routine blood monitoring for people taking clozapine.^16^

In the United Kingdom, routine monitoring of a person's ANC and white blood cell count (WBC) is a mandatory requirement for people taking clozapine. This is mandated by the Medicines and Healthcare products Regulatory Agency (MHRA) and is regulated and managed by the UK clozapine registries (Clozaril® Patient Monitoring Service, CPMS; Denzapine® Monitoring System, DMS; and Zaponex® Treatment Access System, ZTAS).

Clozapine blood monitoring in the United Kingdom follows a traffic light system (Table 1).^17^, ^18^ A person must return a ‘green’ result to start clozapine. Continuation of clozapine depends on weekly ‘green’ results in the first 18 weeks of treatment. After the first 18 weeks, monitoring occurs every 2 weeks for the remainder of the first year and then monthly for the duration of treatment. If an ‘amber’ result is returned, monitoring increases to twice per week until the person's WBC/ANC returns to ‘green’. A ‘red’ result requires immediate discontinuation of clozapine, followed by a management ‘red alert’ protocol (described in Table 1). If two ‘red’ results are recorded on two consecutive days, clozapine must not be restarted, and the person must be registered with the Central Non‐Rechallenge Database. This database is shared across all UK clozapine registries and exists to prevent people with suspected clozapine‐induced agranulocytosis from being re‐exposed to clozapine.

**TABLE 1:** Clozapine blood monitoring in UK practice.^17^, ^18^

**All people taking clozapine are required to undergo WBC and ANC monitoring, as follows**
Before initiating clozapine (pre‐treatment baseline blood test). At least every week for the first 18 weeks of treatment (~4 months). Then, at least at 2‐week intervals until the first year of treatment (between Weeks 18 and 52). After 1 year of treatment with stable WBC/ANC, people taking clozapine are monitored at least at 4‐week intervals. Monitoring must continue throughout treatment and for at least 4 weeks after discontinuation. People taking clozapine and their carers should be warned to contact a doctor if infection develops, especially a fever (≥38°C) or sore throat or flu‐like symptoms. If any occur throughout clozapine treatment, an urgent WBC/ANC should be arranged.			

Thresholds for routine blood monitoring for people taking clozapine			
	Standard thresholds^a^ (×10^9^/L)	DANC/ADAN thresholds^b^ (×10^9^/L)	Clinical actions
Green	WBC ≥ 3.5 and ANC ≥ 2.0	WBC ≥ 3.0 and ANC ≥ 1.5	Continue clozapine treatment and blood monitoring at the standard frequency.
Amber	WBC ≥ 3.0 and <3.5 and/or ANC ≥ 1.5 and <2.0	WBC ≥ 2.5 and <3.0 and/or ANC ≥ 1.0 and <1.5	Continue clozapine treatment with caution. Increase blood monitoring frequency to at least twice per week until results return to ‘green’ status.
Red	WBC < 3.0 and/or ANC < 1.5	WBC < 2.5 and/or ANC < 1.0	Stop clozapine immediately. Monitor for fever or other signs of infection. Follow the ‘red alert’ protocol (as below). If a second blood test on the next day confirms the ‘red’ result, clozapine should not be restarted.
Existing protocol to manage a ‘red alert’: Immediate discontinuation of clozapine is mandatory if a person develops a ‘red’ result, as defined above, and the relevant clozapine monitoring service (e.g., CPMS, DMS or ZTAS) should be informed as soon as possible. People should be monitored for fever, flu‐like symptoms (e.g., sore throat) or other signs of infection. Patients should be reminded to inform their prescriber if any symptoms develop. Daily follow‐up blood samples should continue until counts are in the ‘green’ range. If a second consecutive ‘red’ result (on the following day) occurs, this confirms the red alert, and the person should not be restarted on clozapine. In people with a confirmed ‘red’ result, clozapine should not be restarted. All people with a confirmed ‘red’ should be registered with the Central Non‐Rechallenge Database (CNRD UK or CNRD Ireland) to ensure they are not inadvertently re‐exposed to clozapine from alternative suppliers. Prescribers are encouraged to keep a record of all blood results and to take any steps necessary to prevent the person from being rechallenged with clozapine in error in the future. If the consecutive sample (on the following day) is not ‘red’, the red alert is unconfirmed. Treatment may only be resumed after two ‘green’ results have been obtained. Treatment may be restarted at the normal dose if the break in treatment is less than 48 h; otherwise, the dose may be re‐titrated according to an appropriate dosing schedule. The person must remain off clozapine until the second follow‐up result is received. Following an unconfirmed ‘red’, additional monitoring is needed as a precaution if the follow‐up results are either ‘amber’ or ‘green’ but still low for that person, irrespective of whether clozapine is restarted or not. If the person's ANC falls below 1.0 × 10^9^ /L and/or WBC falls below 2.0 × 10^9^/L, management must be guided by a haematologist. If neutropenic sepsis is suspected, urgent escalation is required including immediate referral to secondary or tertiary care.^19^ All other medications should be reviewed. Consider stopping any drugs that may reduce WBC and/or ANC. If an antipsychotic medication is required, choose a drug with low potential to cause neutropenia, and avoid depot preparations.			

^a^ Standard WBC/ANC thresholds are approved by the MHRA, and these are described in the product SmPCs.^4^, ^5^, ^6^ There are minor differences in the thresholds used to initiate clozapine (i.e., for the pre‐treatment blood test) and monitoring advice between the clozapine registries, CPMS and ZTAS, according to their manuals and factsheets (Table S2). The authors advise that clozapine thresholds and monitoring processes should be harmonized across the SmPC and all registries operating in the United Kingdom.

^b^ DANC/ADAN WBC/ANC thresholds are provided by the United Kingdom's clozapine registries and are not included in the product SmPCs. Please note these may be referred to as BEN (benign ethnic neutropenia) thresholds. Currently, people with confirmed ADAN (also known as BEN/DANC) can have the colour‐coded ranges for blood monitoring decreased by 0.5 × 10^9^/L, relative to standard thresholds (see above DANC/ADAN thresholds). These people should be managed following the normal clozapine protocol but under the DANC/ADAN‐revised thresholds. Some monitoring registries require the agreement of a haematologist to apply the DANC/ADAN‐revised thresholds. The authors of this guideline recommend that all people confirmed to have the Duffy‐null genotype (see Table 2 and Sections 6 and 7 for more detail) should be monitored using DANC/ADAN‐revised thresholds without delay, although we note that this differs with existing UK practice for clozapine prescribing.

The guidance provided in the MHRA‐approved summary of product characteristics (SmPC) for all UK‐licenced clozapine products, Clozaril®, Zaponex® and Denzapine®, is the same. However, the SmPCs do not mention revised thresholds for people with *ACKR1*/*DARC*‐associated neutropenia (described in Section 1.2) or the traffic light system that is used throughout the United Kingdom for WBC/ANC monitoring for people taking clozapine. After reviewing the SmPCs and, where available the literature published by the registries, we have identified differences, which are highlighted in Table S2.

### 1.2 *ACKR1*/*DARC*‐associated neutropenia (ADAN)

Some terms used throughout this guideline are similar and sometimes interchangeable, and these are described in Table 2.

**TABLE 2:** Summary of guideline terminology.

Term type	Term used in this guideline	Equivalent terms
Phenotype	DANC (Duffy‐null associated neutrophil count). ADAN (*ACKR1*/*DARC*‐associated neutropenia).	BEN (benign ethnic neutropenia).
Gene symbol	*ACKR1* (Atypical Chemokine Receptor 1).	*CCBP1* *CD234* *DARC* *DFY* *FY* *GPD* *WBCQ1*
Causal variant and genotype	rs2814778 (c.‐67 T > C). Duffy‐null genotype^a^ is used when referring to people homozygous for the T > C variant (genotype C/C at rs2814778). Duffy‐null variant is used when referring to the T > C variant, rather than the combination of alleles at rs2814778 (i.e., a person's genotype). Duffy‐positive or wild type refers to carriers of a copy of the functional T allele (C/T or T/T at rs2814778).	FY*02 N.01 hg38: chr1:159204893 T > C NG_011626.3: g.5881 T > C NM_002036.3(ACKR1):c.‐67 T > C

^a^ Where ‘Duffy‐null genotype’ is used throughout this guideline, this refers to people who are homozygous for the T > C variant at rs2814778 (genotype C/C). The Duffy‐null genotype at rs2814778 produces a Duffy‐null phenotype exclusively in erythrocytes. Therefore, Duffy antigens (protein products of the *ACKR1* gene) can be expressed in other tissues and cell types in people who are homozygous for the rs2814778 T > C variant. Other variants in the *ACKR1* gene can produce a global Duffy‐null phenotype; however, these are rare and are not explored further in this guideline.

Clozapine prescribers should consider whether a person has ADAN (Table 2).^17^, ^18^, ^20^ People with ADAN have an ANC below the normal range, but they do not have an increased risk of infection or clozapine‐induced agranulocytosis.^20^, ^21^, ^22^, ^23^, ^24^ In the United Kingdom, when ADAN is confirmed by a haematologist, reduced WBC/ANC monitoring thresholds are applied (DANC/ADAN thresholds; Table 1) to help avoid unnecessary clozapine discontinuation and exclusion.^17^, ^18^ However, we note that revised thresholds are not included in the clozapine product SmPCs but instead have been agreed by the clozapine providers and are equivalent across the United Kingdom.

ADAN is caused by a homozygous variant in *ACKR1* (Atypical Chemokine Receptor 1; c.‐67 T > C, rs2814778).^24^, ^25^, ^26^, ^27^ This is known as the Duffy‐null genotype, and the T > C variant at rs2814778 can be referred to as the Duffy‐null variant. The Duffy‐null genotype is prevalent in people of African and Middle Eastern ancestry (approximately 80% and 25%, respectively) but is much less common in people with other ancestries, such as European and East Asian (both around 1%).^20^, ^21^, ^28^, ^29^ Frequency rates for the Duffy‐null variant across different ancestries and datasets are shown in Table 3. The global frequency pattern of the Duffy‐null variant can potentially be explained by selective pressures, as people homozygous for the Duffy‐null variant (i.e., the Duffy‐null genotype) have partial resistance to *Plasmodium vivax* malarial parasite infection.^32^ In 2011, Howes et al.^29^ integrated serological and genetics data to map the global distribution of Duffy blood groups, which confirmed that regions with high prevalence of the Duffy‐null genotype map to historical areas of malaria transmission.^33^

**TABLE 3:** Frequency of the rs2814778 T > C Duffy‐null variant in different ancestries and datasets.

Ancestry	Population frequencies of rs2814778 Duffy‐null variant in *ACKR1* (c.‐67 T > C)	
	All of Us^30^	gnomAD^31^
African	84%	84%
Americas	8%	6%
East Asian	0.05%	0.0%
European	0.5%	0.2%
Middle Eastern	29%	21%
South Asian	0.2%	0.6%

*Note*: Data reported in this table refer to genomic studies and population databases that have been used to provide estimates of variant frequencies. These data are consistent with figures reported in the publication by Howes et al.,^29^ which mapped rates of the Duffy‐null variant and Duffy‐null genotype across global populations. These data^29^ show that the prevalence of the Duffy‐null variant (and Duffy‐null genotype) varies across Africa, with highest rates in Sub‐Saharan Africa. Indeed, in western, central and south‐eastern regions of Sub‐Saharan Africa, ranging from The Gambia to Mozambique, Duffy‐null variant frequencies are estimated to reach fixation at 100%. In All of Us data, the Americas ancestry group includes ‘people who can trace some of their distant ancestors to North, Central, or South America. Many of these people may also have some ancestors who came from other places, like Europe and Africa. People with combinations of Indigenous American genetic ancestry with European and/or African genetic ancestry are included in this category’.^30^ In gnomAD data, the Americas ancestry group is defined as Admixed American ancestry.^31^ We note that people identifying as African American—who predominantly have African ancestry—are likely to have a much higher Duffy‐null variant frequency than reported for the Americas group.^27^, ^34^

The Duffy‐null genotype is strongly associated with low ANC; however, people with the Duffy‐null genotype can present with an ANC in the normal range. For these people, their ANC is likely to be at the lower end of the normal range, with a study reporting the median ANC for Duffy‐null individuals to be 2.8 × 10^9^/L.^34^ Additionally, people with the Duffy‐null genotype are at risk of transient neutropenia, occurring in 33% of Duffy‐null clozapine users over a 6‐month period (65/199), compared with 6% of people with a copy of the functional allele (3/50).^22^ We note that people with a copy of the functional allele (including people heterozygous for the Duffy‐null variant) are unaffected and not at risk of benign neutropenia related to their *ACKR1* genotype.^23^, ^34^

The Duffy‐null variant (rs2814778) disrupts the erythroid GATA1 transcription factor binding site in the promoter region of the *ACKR1* gene.^20^, ^21^ Therefore, people with the Duffy‐null genotype do not express ACKR1 protein in erythrocytes (although other tissues are unaffected).^20^, ^21^ A mechanistic mouse study found that absence of ACKR1 protein in erythrocytes interferes with haematopoiesis, giving rise to neutrophils that preferentially leave peripheral circulation, resulting in apparent neutropenia.^35^ This may explain why people with the Duffy‐null genotype do not have increased risk of infection or morbidity, as their total neutrophil count (when considering neutrophils in both tissues and blood) may be normal.

## 2 LICENCED INDICATIONS

**Table:** 

**Licenced indications for clozapine in the United Kingdom**
**Treatment‐resistant schizophrenia** Clozapine is indicated in treatment‐resistant schizophrenia and in people with schizophrenia who have severe, untreatable neurological adverse reactions to other antipsychotic agents, including atypical antipsychotics. Treatment resistance is defined as a lack of satisfactory clinical improvement despite the use of adequate doses of at least two different antipsychotic agents, including an atypical antipsychotic agent, prescribed for adequate duration. **Psychosis during the course of Parkinson's disease** Clozapine is also indicated in psychotic symptoms occurring during the course of Parkinson's disease, in cases where standard treatment has failed.

*Note*: The exact wording for the currently licenced indications has been extracted from the Summary of Product Characteristics for Clozaril®, approved by the Medicines and Healthcare products Regulatory Agency (MHRA, text revised in 29 August 2023).^4^

## 3 EVIDENCE OVERVIEW

We recently reviewed the evidence for offering *ACKR1*/Duffy‐null genotype testing to people taking clozapine.^36^ Here, we provide a summary.

It is standard UK practice for ADAN to be diagnosed by a haematologist, and this is typically based on clinical criteria after exclusion of other causes of neutropenia.^15^, ^20^ Referral pathways and collaborative links between psychiatry and haematology services vary widely across the United Kingdom, and studies show that ADAN goes frequently undetected in clozapine users.^17^, ^18^, ^24^, ^37^, ^38^, ^39^, ^40^, ^41^ These people—often from ethnic minority backgrounds—are at risk of unnecessary disruption and discontinuation of their clozapine therapy.

It is well recognized that clozapine is underused and its initiation delayed with studies citing an average of 8 years to treatment commencement, which does not align with NICE guidelines.^42^, ^43^ Additionally, ADAN diagnoses are also delayed, with one study from a large London‐based mental health service reporting an average of 8.8 years.^18^ For people with the Duffy‐null genotype, prompt application of revised monitoring thresholds can substantially reduce treatment disruption and help to avoid unnecessary clozapine discontinuation.^39^

For people who need clozapine, treatment discontinuation and/or exclusion is linked to adverse outcomes, including hospitalization, relapse and suicide,^44^, ^45^, ^46^ and reinitiating clozapine after a period of discontinuation can in some cases require higher doses than usual.^47^ In the United Kingdom, if a person is registered with the Central Non‐Rechallenge Database, restarting clozapine can only be performed off‐license.^16^, ^18^, ^38^ Moreover, operational processes differ between clozapine registries/suppliers, and decisions about clozapine rechallenge are case‐specific and subject to regulatory and product information constraints. These factors can jeopardize restarting of clozapine, even when it is appropriately indicated (such as following a diagnosis of ADAN). It is therefore crucial that clozapine discontinuation—unless clinically necessary—is avoided wherever possible.

*ACKR1*/Duffy‐null genotype testing provides an opportunity to promptly identify people with the Duffy‐null genotype (and, therefore, people who have or are at risk of ADAN), who should be monitored with revised DANC/ADAN thresholds (Table 1). The Royal College of Psychiatrists has highlighted the potential benefits of *ACKR1*/Duffy‐null genotype testing in a report reviewing genetic testing in mental health,^48^ and the Maudsley Prescribing Guidelines in Psychiatry, widely used in the United Kingdom and internationally, recommend *ACKR1*/Duffy‐null genotype testing as part of standard clozapine therapy.^40^, ^49^ Currently, *ACKR1*/Duffy‐null genotype testing for people taking clozapine is not routinely offered within the United Kingdom's National Health Service (NHS), though the test is available via commercial providers.^40^

In this CERSI‐PGx guideline, we provide the first evidence‐based framework to deliver *ACKR1*/Duffy‐null genotype testing at scale for clozapine users in the United Kingdom's NHS. We propose a model for equitable access and integration of the test within the existing UK care pathway. Following evaluations, *ACKR1*/Duffy‐null genotype testing for people taking clozapine may be considered in other countries.

## 4 RECOMMENDED INDICATIONS FOR PHARMACOGENETIC TESTING

These recommendations apply to all people taking clozapine for any indication in the United Kingdom. They are appropriate for people of all ages, although the UK clozapine licence is for people 16 years and older.

*ACKR1*/Duffy‐null genotype testing only needs to be conducted once. We recommend three eligibility criteria for testing.

**Criterion 1: pre‐emptive testing for people starting clozapine**
  - All people starting clozapine should be offered *ACKR1*/Duffy‐null genotype testing. Testing should not be restricted to groups based on self‐reported ethnicity, as this can be an unreliable measure of genetic ancestry.^50^, ^51^
  - Pre‐emptive testing ensures revised thresholds for ADAN/DANC are applied as early as possible.
  - People who have discontinued clozapine and are re‐starting it should be offered the *ACKR1*/Duffy‐null genotype test, unless they have been tested before.

**Criterion 2: testing for people registered in the Central Non‐Rechallenge Database**
  - People with ADAN can be unnecessarily registered in the Central Non‐Rechallenge Database after being incorrectly classified with clozapine‐induced neutropenia or agranulocytosis.^18^, ^38^, ^41^
  - This criterion will identify people with the Duffy‐null genotype (and, therefore, people who have or are at risk of ADAN) in the Central Non‐Rechallenge Database, who may be considered for clozapine rechallenge.

**Criterion 3: reactive testing for people returning an ‘amber’ or ‘red’ blood result**
  - People who develop ‘amber’ or ‘red’ blood results (Table 1) are at risk of disruption to/discontinuation of clozapine therapy.
  - These people should be considered for testing unless tested via the other two criteria.

The availability of *ACKR1*/Duffy‐null genotype testing may vary in time, indication and geography. In the absence of available information or testing, current best practice guidelines should be followed. In the longer term, the most important route to this test will be under Criterion 1. Over time, testing under Criteria 2 and 3 may become less frequent as people starting clozapine are tested pre‐emptively.

## 5 INTEGRATING PHARMACOGENETIC TESTING INTO EXISTING CLINICAL PATHWAYS

People taking clozapine in the United Kingdom (for any indication) must be registered with a clozapine registry: Clozaril® Patient Monitoring Service, CPMS; Denzapine® Monitoring System, DMS; or Zaponex® Treatment Access System, ZTAS. The registries should adhere to information provided in the SmPCs for clozapine‐containing medicinal products (Table 1). Therefore, recommendations in this guideline should be generalisable across the United Kingdom.

The clozapine prescriber (usually a psychiatrist) should request the *ACKR1*/Duffy‐null genotyping test. Provision of the test should not change or hinder the clozapine treatment pathway. While waiting for the test result, clozapine therapy should continue (or be stopped or withheld) according to standard protocol. The test should be voluntary and should not delay the initiation of clozapine therapy.

Under pre‐emptive testing (Criterion 1), the sample for the test may be obtained during the standard pre‐treatment blood test, or as soon as possible thereafter as part of routine monitoring. Under Criterion 2 (people registered with the Central Non‐Rechallenge Database), an additional blood sample may be required as eligible people may not be undergoing routine blood monitoring. Under Criterion 3 (reactive testing following an ‘amber’ or ‘red’ blood result), a sample should be obtained as soon as possible at the next scheduled blood test.

*ACKR1*/Duffy‐null genotyping test results should be recorded in the electronic health record using a relevant SNOMED CT ID (Systematized Nomenclature of Medicine—Clinical Terms Identifier), and we suggest new SNOMED CT IDs should be created to accurately record test results with respect to the rs2814778 variant. Ideally, test results should be recorded by the clozapine registry using identical SNOMED CT ID codes. However, we are aware this may require adaptations to existing registry systems, and we recommend that delays to system changes should not hinder the use of the test or application of revised thresholds. In the immediate future, test results may be recorded in line with existing system requirements (such as being captured in free text fields), to ensure quick application of revised thresholds for people with the Duffy‐null genotype. Additionally, when the NHS Unified Genomic Record becomes widely available, as described in the NHS 10‐year Health Plan for England,^52^ genotyping results may be stored for every person in a standardized and easily accessible format. When the test result is ready, the person should be informed, and educational materials should be developed to support these conversations.

To realize the benefits of *ACKR1*/Duffy‐null genotype testing, the existing UK clozapine monitoring pathway should be amended for people with the Duffy‐null genotype. Pre‐emptive testing (Criterion 1) will identify people with the Duffy‐null genotype who have a WBC/ANC in the normal range. These people should be monitored using DANC/ADAN‐revised thresholds, currently reserved for people with a clinical diagnosis of ADAN (Table 1). Evidence shows that people with the Duffy‐null genotype are significantly more likely to have a lower ANC than those with a copy of the functional allele (T/T or T/C at rs2814778), although they may initially present with an ANC in the normal range (>2.0 × 10^9^/L).^22^, ^23^ Moreover, people with the Duffy‐null genotype are more likely to develop transient neutropenia, meaning they may experience disruption to (and, ultimately, discontinuation of) their clozapine treatment.^22^, ^23^ Therefore, we recommend that all people with the Duffy‐null genotype should be monitored using revised thresholds for ADAN and DANC as soon as possible to support fair access to clozapine.

## 6 WHICH GENE(S), VARIANTS, TURNAROUND TIME

The genotyping test is for the rs2814778 variant (c.‐67 T > C) in the *ACKR1* gene. Homozygous carriers of the variant (C/C at rs2814778) have the Duffy‐null genotype, which is the causal genotype for ADAN and DANC.

People who have a copy of the functional allele (genotype C/T or T/T at rs2814778 in the *ACKR1* gene; also known as Duffy‐positive or wild type) are not affected and do not need to be assessed for ADAN.

The recommended laboratory turnaround time is 1 week. This is the optimal turnaround time given the existing clinical pathway, which requires weekly full blood counts during the first 18 weeks of clozapine therapy, when neutropenia is more likely.

## 7 CLINICAL ACTIONS BASED ON GENOTYPE

**FIGURE 1:**
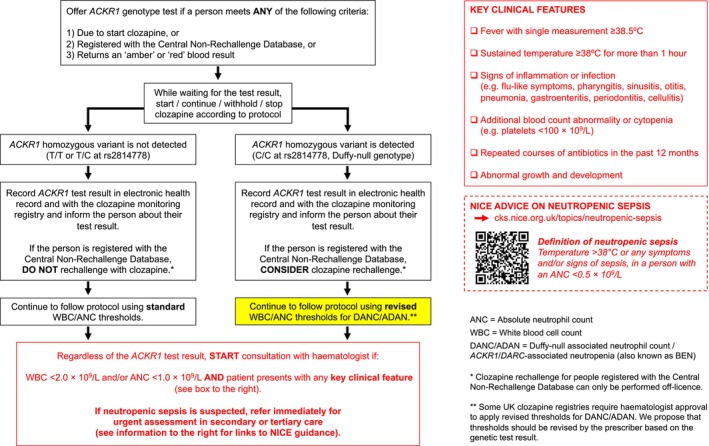
Integrating *ACKR1*/Duffy‐null genotype testing into the existing UK clinical pathway for clozapine.

Clinical actions are described below and summarized in Figure 1.

### 7.1 Actions when the *ACKR1* homozygous C/C variant at rs2814778 (Duffy‐null genotype) is detected

- Record the test result in the person's electronic health record and inform the clozapine registry to prevent unnecessary repetition of the test. At present, SCTID: 115835008 is available to record the Duffy‐null phenotype (Fy[a‐b‐]). New SNOMED CT IDs are required to record the Duffy‐null genotype with respect to the rs2814778 variant.
- Apply revised WBC/ANC thresholds for DANC/ADAN (see Table 1) as soon as possible. Some UK clozapine registries require haematology authorization to apply the DANC/ADAN‐revised thresholds. We recommend that a test result confirming the Duffy‐null genotype is sufficient to apply DANC/ADAN‐revised thresholds without delay. This is supported by the European Guidelines on Diagnosis and Management of Neutropenia in Adults and Children, which advise that extensive work‐up is not required for people with the Duffy‐null genotype.^15^
- For people registered with the Central Non‐Rechallenge Database (i.e., tested under Criterion 2), clozapine rechallenge under DANC/ADAN‐revised thresholds should be considered by the treating physician and clinical team. At present, it is not possible to remove a person from the Central Non‐Rechallenge Database; however, a note may be added to the database system stating that the person has been rechallenged.
- Communicate the result of the test to the patient (i.e., the Duffy‐null genotype), as well as the rationale for changes to their monitoring thresholds and clozapine treatment.
- Follow standard UK protocol for clozapine while applying revised monitoring thresholds for DANC/ADAN (Table 1).
- If the person returns a WBC < 2.0 × 10^9^/L and/or ANC < 1.0 × 10^9^/L and if they present with any key clinical feature (Figure 1), start a consultation with a haematologist who can advise about appropriate management and treatment. If neutropenic sepsis is suspected (defined as a temperature of greater than 38°C or any symptoms and/or signs of sepsis, in a person with an ANC < 0.5 × 10^9^/L), refer immediately for urgent assessment in secondary or tertiary care. A QR code and hyperlink to further NICE information about neutropenic sepsis are provided in Figure 1.^19^
- If for any reason (regardless of their *ACKR1* test result), the person is unable to take/continue clozapine, follow local or national guidelines for treatment with an alternative medication.^49^

### 7.2 Actions when the *ACKR1* homozygous C/C variant is NOT detected (carriers of a copy of the functional allele, C/T or T/T at rs2814778)

- Record the test result in the person's electronic health record (as noted above, new SNOMED CT IDs are required to capture genetic variation at rs2814778) and inform the clozapine registry to prevent unnecessary repetition of the test.
- For people registered with the Central Non‐Rechallenge Database, clozapine rechallenge should not be considered based on the test result (as these people are not at risk of benign neutropenia with respect to their *ACKR1* genotype). If clozapine rechallenge is considered appropriate for another clinical reason, then a note may be added to the database system stating that the person has been rechallenged.
- If the person is starting or already taking clozapine, continue to apply standard monitoring thresholds and follow the standard UK protocol for clozapine (see Table 1).
- Communicate the result of the *ACKR1* genotype test to the patient.
- If the person returns a WBC < 2.0 × 10^9^/L and/or ANC < 1.0 × 10^9^/L and if they present with any key clinical feature (Figure 1), start a consultation with a haematologist who can advise about appropriate management and treatment. If neutropenic sepsis is suspected (defined as a temperature of greater than 38°C or any symptoms and/or signs of sepsis, in a person with an ANC < 0.5 × 10^9^/L), refer immediately for urgent assessment in secondary or tertiary care. A QR code and hyperlink to further NICE information about neutropenic sepsis are provided in Figure 1.^19^
- If for any reason (regardless of their *ACKR1* test result), the person is unable to take/continue clozapine, follow local or national guidelines for treatment with an alternative medication.^49^

Although this guideline is grounded in the latest evidence in the field, it cannot account for all individual factors relevant to patient care. Therefore, prescribers must conduct a thorough assessment of each patient's risk–benefit profile, ensuring that therapy is optimized to maximize benefits and minimize potential harms.

## 8 OTHER PHARMACOGENETICS GUIDELINES

In this section, we have summarized prescribing recommendations provided by international pharmacogenomics consortia.

To the best of our knowledge, existing pharmacogenomics guidelines do not cover the gene‐drug pair, *ACKR1*‐clozapine. We propose that *ACKR1* should be considered by relevant guideline‐making bodies due to its clear relevance for clozapine prescribing.

### 8.1 Clinical Pharmacogenetics Implementation Consortium guidelines

At the time of writing, the Clinical Pharmacogenetics Implementation Consortium (CPIC) does not cover the *ACKR1* gene for clozapine in any of their guidelines.

CPIC has given provisional status for potential clinically relevant interactions with clozapine to several genes, including *CYP2D6*, *HTR2C*, *MC4R* and *ANKK1*.^53^ However, the level of evidence at present is not high for these gene‐drug pairs in relation to clozapine.

### 8.2 Dutch Pharmacogenetics Working Group guidelines

At the time of writing, the Dutch Pharmacogenetics Working Group (DPWG) does not cover the *ACKR1* gene for clozapine in any of their guidelines.

The DPWG highlights that clozapine is mainly metabolized by CYP1A2 to *N*‐desmethylclozapine (norclozapine), which is an active metabolite.^54^ The contribution of other CYP450 enzymes, including CYP2D6, to clozapine metabolism is considered minor.^55^ Most available studies show no increase in adverse effects, including the development of agranulocytosis, in CYP2D6 poor metabolizers, intermediate metabolizers, and ultra‐rapid metabolizers. Therefore, the DPWG does not recommend dose‐adjustment based on *CYP2D6* genetic variation. Similar conclusions have also been reached for *CYP1A2*, and at present, no actionable gene‐drug interactions for clozapine are reported.^54^ It is also important to note that since late 2024, the CYP1A2*1C allele has been retired due to evidence not meeting PharmVar minimum standards.^56^, ^57^

### 8.3 Other guidelines

Spain's national healthcare system recently approved a pharmacogenomics testing catalogue.^58^ The catalogue includes 12 genes that relate to the prescribing of 22 drugs; however, clozapine and *ACKR1* are not included at present.

The US Food and Drug Administration (FDA) clozapine drug label contains a recommendation that dose reduction may be required in people who are CYP2D6 poor metabolizers, as well as dose adjustment recommendations for people also taking CYP1A2 inducers and/or inhibitors.^59^ However, the US FDA does not provide recommendations for *ACKR1*/Duffy‐null genotype testing in relation to clozapine.^59^

## 9 HEALTH ECONOMIC EVALUATION

The health economic evidence for *ACKR1*/Duffy‐null genotype testing is summarized from our previous analysis.^36^ No other economic evaluations have been identified.

For the management of treatment‐resistant schizophrenia, clozapine is economically dominant (i.e., more effective and less costly) compared with other antipsychotic drugs.^60^, ^61^, ^62^ Therefore, the economic case for *ACKR1*/Duffy‐null genotype testing depends on net costs of treatment and genotyping. From the perspective of the NHS (for the whole of the United Kingdom), we provide estimates for the economic impact during the first year of testing, including conservative and anti‐conservative calculations of savings and costs associated with clozapine treatment and the price of testing.^36^ These value ranges (conservative and anti‐conservative) take into consideration the uncertainty surrounding the true cost saving associated with clozapine treatment to the UK healthcare system, as well as the variable price of the genetic test.

Annual savings associated with clozapine therapy were £3783 (conservative)^63^ and £6864 (anti‐conservative),^62^ and genetic test prices were £169 (conservative, based on the price of a targeted sequencing approach) and £60 (anti‐conservative, based on the price of a similar assay for single nucleotide variants in the *DPYD* gene). Data were derived from published sources and NHS laboratories and were inflated to 2025 prices. The analysis estimated the financial implications related to each of the three eligibility criteria (Table 4). Considering all criteria combined, total cost savings across the United Kingdom in the first year of testing were estimated to lie between £42 732 (conservative) and £727 990 (anti‐conservative).^36^

**TABLE 4:** Health economic evaluation of *ACKR1*/Duffy‐null genotype testing for people taking clozapine in the UK (summarized from Murtough et al.^36^).

Criteria	Parameters included in the analysis	Annual costs/savings across the United Kingdom
Criterion 1: Pre‐emptive testing for people starting clozapine	A UK population estimate for 16 to 64‐year‐olds of 43 295 000 people.^63^ Annual schizophrenia incidence rate for 16 to 64‐year‐olds in England (which was assumed to be representative of the United Kingdom) of 15.2 per 100 000 person‐years.^64^ An estimated test detection rate (i.e., proportion of people returning the Duffy‐null genotype) of ~3.85% for the UK population.^36^ Figure estimated from 2021 UK four nations' censuses self‐reported ethnicity data^65^, ^66^, ^67^ and published Duffy‐null variant frequencies in diverse world populations.^28^ Accounting for 30% of people with schizophrenia are treatment resistant.^8^	**Conservative**: −£46 108 (a cost) **Anti‐conservative**: £403 269 (saving)
Criterion 2: Testing for people registered in the Central Non‐Rechallenge Database	Assumption that people registered in the past 3 years may be tested during the first year of available *ACKR1* testing, totalling 588 people.^38^ A test detection rate (i.e., proportion of people returning the Duffy‐null genotype) of 8.54% for people registered in the Central Non‐Rechallenge Database, given the over‐representation of people with Black, African, and/or Caribbean ethnicity in the database, and calculated from published data.^38^	**Conservative**: £90 592 (saving) **Anti‐conservative**: £309 397 (saving)
Criterion 3: Reactive testing for people returning an ‘amber’ or ‘red’ blood result	Using UK population figures, schizophrenia incidence rate and test detection rate as described for Criterion 1. Assumption that neutropenia (defined as a UK ‘red’ result, ANC < 1.5 × 10^9^/L) occurs in 3.8% of people taking clozapine.^14^	**Conservative**: −£1752 (a cost) **Anti‐conservative**: £15 324 (saving)
**All Criteria Combined**	These figures report the net saving (or cost) during the first year of testing for all eligible people taking clozapine across the United Kingdom. In subsequent years, it is likely that costs will decrease while savings for people already tested will continue to accrue each year.	**Conservative**: £42 732 (saving) **Anti‐conservative**: £727 990 (saving)

In subsequent years of testing, it is likely that testing costs will decrease whereas savings for people already tested will continue to accrue. As new clozapine starters are gradually tested under Criterion 1, the proportion of people requiring reactive testing under Criteria 2 and 3 will decrease over time. Genetic testing costs may also decrease as the demand for testing increases. An additional opportunity could be to incorporate the *ACKR1* test into a pharmacogenomics testing panel, where a person would need to be tested once to inform prescribing across multiple health specialties over a lifetime.

## 10 REGULATORY CONSIDERATIONS

### 10.1 Summary of product characteristics (SmPC) and the UK monitoring system

The SmPC of licenced medicinal products containing clozapine,^4^, ^5^, ^6^ in line with European regulatory documents, states that blood monitoring should be carried out in accordance with national specific official recommendations. UK regulations mandate ANC and WBC monitoring as described in Table 1.

In the United Kingdom, there are three mandatory national patient monitoring services (also referred to as the clozapine registries) for the management of agranulocytosis risk associated with clozapine use. These services are managed by the companies that manufacture and supply the clozapine. The monitoring services are available 24 h a day and provide centralized WBC/ANC monitoring. For further guidance and information, contact details for the monitoring services can be found here:

- Clozaril® Patient Monitoring Service (CPMS; https://www.clozaril.co.uk/scrLogon.asp)
- Denzapine® Monitoring System (DMS; https://www.denzapine.ie/)
- Zaponex® Treatment Access System (ZTAS; https://www.ztas.co.uk/)

People taking clozapine, prescribing physicians and nominated pharmacists should be registered with the relevant national patient monitoring service. Drug supply is restricted to hospital and community pharmacies registered with these services.

To ensure safety, at any one‐time people should be prescribed one brand of clozapine and registered with the monitoring service connected to that brand. Prescribers and pharmacists should adhere to brand prescribing and dispensing of clozapine to prevent disruption to blood monitoring that may be caused following brand switching. The SmPCs for all clozapine containing medicinal products licenced in the United Kingdom recommends special consideration for people with BEN (i.e., ADAN or DANC), in whom clozapine may only be started with the agreement of a haematologist. The current SmPCs do not include any information on *ACKR1*/Duffy‐null genotype testing.

The SmPC also includes warnings about potential risk of cardiotoxicity (myocarditis and fatal cardiomyopathy).^4^, ^5^, ^6^ However, we note there is no relationship between risk of cardiac adverse events with clozapine and *ACKR1*.

### 10.2 MHRA drug safety updates

On 26 August 2020, the MHRA issued a drug safety update recommending therapeutic drug monitoring (measuring clozapine and norclozapine in blood) in certain clinical situations.^68^ These situations include when a person ceases smoking or switches to e‐cigarettes; when concomitant medicines may interact to increase clozapine blood levels; where poor or reduced clozapine metabolism is suspected; when a person has pneumonia or other serious infection; and when toxicity is suspected.^68^

### 10.3 NICE guidance

The National Institute for Health and Care Excellence (NICE) guidance on ‘psychosis and schizophrenia in adults’ recommends clozapine use after failure of two antipsychotic drugs.^2^ It remarks that service providers, including general practitioners, community health services, mental health services and hospitals should have in place procedures and protocols to monitor the prescribing of clozapine. However, it does not mention the potential for adverse drug reactions associated with clozapine use nor genetic predictors such as the rs2814778 T > C variant in *ACKR1*. In subsequent guidance on Parkinson's disease, NICE recommends clozapine use (at lower doses than for treatment‐resistant schizophrenia) together with adequate monitoring in people with Parkinson's disease affected by hallucinations and delusions.^69^ However, as above, there is no mention of potential adverse drug reactions nor genetic predictors.

### 10.4 International regulations and guidance

The European Guidelines on Diagnosis and Management of Neutropenia in Adults and Children^15^ underlines that the definition of neutropenia varies in relation to ancestry and age. It reminds that people with African and Middle Eastern ancestry can have ANCs in a lower range (0.5 to 1.5 × 10^9^/L) in comparison to people with European ancestry. Their guideline states that this is due to the Duffy‐null genotype/homozygous variant at rs2814778 in *ACKR1*.

Regulation of clozapine monitoring in the United States has undergone several changes over the past 10 years. In October 2015, the FDA lowered the minimum ANC threshold for clozapine discontinuation by 0.5 × 10^9^/L, relative to the UK thresholds, and removed the requirement for WBC monitoring.^38^ During this time, the FDA released a Risk Evaluation and Mitigation Strategy (REMS) programme to further improve monitoring and management.^18^, ^38^, ^70^ These changes aimed to reduce unnecessary discontinuation of vital clozapine treatment. Since then, the FDA label for clozapine has recommended revised ANC thresholds for people with documented ‘benign ethnic neutropenia’ (synonym for ADAN, Table 2), with the normal ANC threshold being >1.0 × 10^9^/L.^59^ In the United States, monitoring was performed more frequently than in the United Kingdom, requiring ANC testing at least weekly for 6 months, at least fortnightly for the next 6 months and at least at monthly intervals after 1 year.^59^ In 2022, Oloyede et al.^38^ produced a modelling study that investigated what might happen if the United Kingdom followed similar policy changes to the United States. They found that implementing FDA monitoring criteria would significantly reduce clozapine discontinuation in the United Kingdom and would improve mental and physical health outcomes.^38^ In 2025, the FDA confirmed further changes with the removal of the REMS mandatory monitoring system for clozapine.^70^ Therefore, there is no longer a mandatory blood monitoring requirement in the United States for people taking clozapine, although monitoring according to the drug label is recommended. This means that a person will not be prohibited from accessing a clozapine prescription if they do not have a recent ANC result.

In September 2025, the European Medicines Agency (EMA) issued revised monitoring recommendations for clozapine.^71^ The EMA removed the WBC monitoring requirement, indicating it is sufficient to measure ANC only. The ANC thresholds were revised as follows: mild (ANC: 1000–1500/mm^3^), moderate (ANC: 500–999/mm^3^) and severe neutropenia (ANC < 500/mm^3^). Initiation of clozapine is recommended for people with an ANC ≥ 1.5 × 10^9^/L, and for people with confirmed ‘benign ethnic neutropenia’ (i.e., ADAN; Table 2), the ANC must be ≥1.0 × 10^9^/L. Clozapine must be stopped (and rechallenge not attempted) in people with an ANC < 1.0 × 10^9^/L (or <0.5 × 10^9^/L in people with confirmed ‘benign ethnic neutropenia’). The EMA have also provided new recommendations that blood monitoring should take place weekly during the first 18 weeks of treatment, then monthly over the following 34 weeks (i.e., until completion of the first year of treatment). In absence of any history of neutropenia during the first year of treatment, ANC monitoring can be reduced to once every 12 weeks. In the absence of any history of neutropenia during the first 2 years of treatment, ANC may be tested once a year.^71^

Finally, in 2025, it was announced that the MHRA is planning to review blood monitoring requirements for clozapine.^72^ If clozapine monitoring guidelines change in the United Kingdom, this guideline will be adapted to map to new monitoring thresholds and protocols. As with recommendations in the United States and Europe, monitoring thresholds for ADAN should be revised so that they are lower than standard thresholds.

## 11 OTHER CONSIDERATIONS

A genotyping test for the rs2814778 variant in *ACKR1* is the preferred method for identifying people with ADAN. However, there may be instances when genotype testing is unavailable. Here, serological testing for a person's Duffy blood group may be considered as an alternative testing method. People who are negative for Duffy antigens (protein products of the *ACKR1* gene) on the surface of their erythrocytes may be assumed to be at risk of ADAN, and the clozapine prescriber may consider monitoring using DANC/ADAN‐revised thresholds if the person is confirmed to have ADAN. If serological testing is used (in lieu of genotype testing), haematology input should be sought to aid with interpretation of a person's neutrophil count in light of the serological test result. This is important because it is only the T > C variant at rs2814778 in *ACKR1* that has been robustly associated with low neutrophil counts and ADAN (to date in the literature),^24^, ^25^, ^26^, ^27^ rather than other variants that may also cause a Duffy‐null phenotype.

We previously estimated the prevalence rate of the Duffy‐null genotype in the United Kingdom to be 3.85%.^36^ In the absence of real‐world genetic testing data, we approximated overall prevalence using ethnicity data self‐reported in the 2021 Censuses of the four UK nations alongside Duffy‐null variant frequencies. In reality, Duffy‐null prevalence will vary across the United Kingdom depending on the genetic ancestry of a given local population. For instance, in areas with a higher proportion of people with African or Middle Eastern genetic ancestry, there will be more people with the Duffy‐null genotype. If the *ACKR1* genotyping test is not nationally adopted in the United Kingdom but is instead offered locally by individual NHS Integrated Care Boards, local leaders may attempt to determine the need for *ACKR1* testing by considering the ethnicity make‐up of their local population. We stress that self‐reported ethnicity is not an accurate or suitable way to decide who should be offered *ACKR1* genetic testing.^50^, ^51^ Indeed, evidence shows that people identifying as ‘White’ (who may be assumed to have European genetic ancestry) can have the Duffy‐null genotype.^23^, ^40^ Therefore, we advise that genetic testing should be offered irrespective of the ethnicity make‐up of a given local population, even if the test is considered for approval on a local basis.

## 12 RESEARCH RECOMMENDATIONS

Although *ACKR1*/Duffy‐null genotype testing for the rs2814778 variant has a strong evidence base, future research is needed to ensure continued progress and improvement in the testing programme.

Research into mechanisms underpinning clozapine's effectiveness for treatment‐resistant schizophrenia, compared to other antipsychotics, is needed. Moreover, research into alternative therapies for people who cannot be rechallenged with clozapine would lead to a more effective treatment pathway.

To increase access to clozapine, research into mechanisms of clozapine‐induced neutropenia and agranulocytosis is needed. If these can be understood, targeted interventions may be used to predict and/or reduce the incidence and severity of these events during clozapine therapy. For instance, some HLA genotypes have been associated with risk of clozapine‐induced neutropenia and agranulocytosis.^73^, ^74^ These findings are promising, though their predictive accuracy requires future validation.

To clarify conflicting pharmacogenomic recommendations for clozapine by the FDA, CPIC and DPWG, larger and more detailed studies are needed. In particular, studies should seek to clarify the effects of genomic variation in *CYP2D6*, *CYP1A2* and *CYP3A4* (as enzymes known to be involved in clozapine metabolism), as well as less studied genes such as *CYP3A5* or *NFIB* that may also contribute to clozapine metabolism and drug exposure.^75^, ^76^, ^77^

*ACKR1*/Duffy‐null genotype testing may support prescribing of other drugs associated with neutropenia and agranulocytosis. This type of testing could also facilitate access to oncology clinical trials where remaining above a minimum ANC threshold is often part of the eligibility criteria.^78^ Moreover, further research is needed to characterize other *ACKR1* genetic variants (including rare variants) that may be associated with benign neutropenia.

Lastly, we have drafted these guidelines based on best available evidence and informed by expert opinion. If our recommendations are implemented in UK NHS services, we anticipate adjustments may be required after testing is rolled out. Close attention should be made towards the costs and savings of the testing programme, diagnostic and test eligibility parameters, and the blood monitoring programme for people with the Duffy‐null genotype.

## AUTHOR CONTRIBUTIONS

Elvira Bramon chaired the writing committee. *Conceptualization*: Stephen Murtough, Cinzia Dello Russo, Munir Pirmohamed and Elvira Bramon. *Methodology*: Stephen Murtough, Cinzia Dello Russo, Munir Pirmohamed and Elvira Bramon. *Investigation*: Stephen Murtough, Oriella Stellakis, Daisy Mills, Lauren Varney, Cinzia Dello Russo, Elvira Bramon contributed to Sections 1 and 3. All authors contributed to Sections 4, 5 and 7. Stephen Murtough, Oriella Stellakis, Huajie Jin, Elvira Bramon and Dyfrig A. Hughes contributed to Section 9. Cinzia Dello Russo contributed to Sections 8 and 10. Stephen Murtough, Cinzia Dello Russo, Munir Pirmohamed and Elvira Bramon contributed to Section 12. *Resources and funding acquisition*: Elvira Bramon and Munir Pirmohamed. *Writing—original draft*: Stephen Murtough, Oriella Stellakis, Cinzia Dello Russo, Dyfrig A. Hughes, Munir Pirmohamed and Elvira Bramon. *Writing—review and editing*: All authors. Stephen Murtough, Cinzia Dello Russo, Munir Pirmohamed and Elvira Bramon revised the guideline according to feedback received after the consultation process. All authors approved the revised version of the guideline and comments provided in Table S3.

## CONFLICT OF INTEREST STATEMENT

Conflict of interest statements for all authors are shown in Table S1.

## Supporting information


**Table S1:** Guideline committee affiliations, expertise, and conflicts of interest.
**Table S2:** Differences between the SmPC for clozapine and ZTAS and CPMS guidance.
**Table S3:** External Consultation Comments and Responses.

## Data Availability

The data that support this guideline are publicly available and fully referenced.
