# Supplementary material for: ACKR1/Duffy‐null genotype testing for clozapine: A guideline developed by the UK Centre of Excellence in Regulatory Science and Innovation in Pharmacogenomics (CERSI‐PGx)
Source: Br J Clin Pharmacol. 2026 May 13;92(7):1977–90. doi: 10.1002/bcp.70576 (PMC13304278; doi:10.1002/bcp.70576)
Supplement: Supplementary file 1 — Table S1: Guideline committee affiliations, expertise, and conflicts of interest. Table S2: Differences between the SmPC for clozapine and ZTAS and CPMS guidance. Table S3: External Consultation Comments and Responses. [file BCP-92-1977-s001.docx]

***ACKR1*/Duffy-null genotype testing for clozapine: a guideline developed by the UK Centre of Excellence in Regulatory Science and Innovation in Pharmacogenomics (CERSI-PGx)**

**AUTHOR INFORMATION**

Stephen Murtough^1^*, Oriella Stellakis^1^*, Daisy Mills^1^*, Blanca Bjourson^2^, Vicky Chaplin^3^, Dharmisha Chauhan^4^, Bev Chipp^5^, Marius Cotic^1^, Jana de Villiers^6,7^, Olubanké Dzahini^8,9^, Frances Elmslie^10^, Katie Evans^11^, Shreyans Gandhi^12^, Dyfrig A Hughes^13^, Huajie Jin^14^, Daniele Panconesi^1^, Anna Skowronska^15^, Sanjay M Sisodiya^16^, Ed Silva^17^, Vicky Stinton^18^, Sara Stuart-Smith^12^, Sarah Tarrant^19^, David Taylor^8,9^, Lauren Varney^1^, James TR Walters^20^, Michelle Wood^11,21^, Jessica Woodley^15^, Cinzia Dello Russo^22,23,§^, Munir Pirmohamed^22,24,§^, and Elvira Bramon^1,25,§^

* Joint first authors.

^§^ Corresponding authors.

**^1^** Division of Psychiatry, University College London, London, UK

^2^ Belfast Health and Social Care Trust, Belfast, UK.

^3^ Genomics Unit, National Health Service England, London, UK.

^4^ North Thames Genomic Medicine Service Alliance, London, UK.

^5^ The Side-By-Side Network, London, UK.

^6^ The Royal College of Psychiatrists, London, UK.

^7^ High Secure Intellectual Disability Service for Scotland and Northern Ireland, Carstairs, UK.

^8^ Pharmacy Department, South London and Maudsley NHS Foundation Trust, London, UK.

^9^ Institute of Pharmaceutical Science, King’s College London, London, UK.

^10^ South East Genomic Medicine Service Alliance, London, UK.

^11^ Cardiff and Vale University Health Board, Cardiff, UK.

^12^ Department of Haematology, King’s College Hospital NHS Foundation Trust, London, UK.

^13^ Centre for Health Economics and Medicines Evaluation, North Wales Medical School, Bangor University, Bangor, UK.

^14^ King’s Health Economics, Institute of Psychiatry, Psychology & Neuroscience, King’s College London, London, UK.

^15^ Birmingham Women’s and Children’s NHS Foundation Trust, Birmingham, UK.

^16^ Research Department of Epilepsy, Queen Square Institute of Neurology, University College London, London, UK.

^17^ Specialist Community Forensic Team for people with Learning Disability and Autism, Hollins Park Hospital, Warrington, UK.

^18^ North West Genomic Laboratory Hub, Manchester University NHS Foundation Trust, Manchester, UK.

^19^ NHS Blood and Transplant, Bristol, UK.

^20^ Centre for Neuropsychiatric Genetics and Genomics, Division of Psychological Medicine and Clinical Neurosciences, Cardiff University, Cardiff, UK.

^21^ All Wales Medical Genomics Service, Cardiff, UK.

^22^ Department of Pharmacology and Therapeutics, Institute of Systems, Molecular and Integrative Biology, University of Liverpool, Liverpool, UK.

^23^ Department of Translational Medicine and Surgery, Section of Pharmacology, Università Cattolica del Sacro Cuore - Fondazione Policlinico Universitario A. Gemelli, IRCCS, Rome, Italy.

^24^ The Wolfson Centre for Personalised Medicine, Centre for Drug Safety Science, University of Liverpool, Liverpool, UK.

^25^ North London NHS Foundation Trust, London, UK.

**SUPPLEMENTARY INFORMATION**

**METHODOLOGY**

**The writing committee**

This guideline (*ACKR1*/Duffy-null genotype testing for clozapine: a guideline developed by the UK Centre of Excellence in Regulatory Science and Innovation in Pharmacogenomics (CERSI-PGx)) has been co-developed by a multidisciplinary team with expertise in psychiatry (including adult, children and young people, and forensic sub-specialities), mental health pharmacy, haematology, neurology, health economics, genomics, and clinical laboratory science. We have also had active representation from the [Side-By-Side Network](https://sidebysidenetwork.org/) (see below).

The writing committee includes members from England, Wales, Scotland, and Northern Ireland. Full disclosure of affiliations, competences, and conflicts of interest are provided below in Supplementary Table 1. The writing committee held their first online meeting on 24^th^ June 2025, and they subsequently met a further three times in 2025 (24^th^ July, 20^th^ August, and 2^nd^ October) to finalise the guideline draft for consultation, with written contributions from members provided throughout this period. Smaller group meetings were held *ad hoc* to discuss specific topics of the guideline.

**Standardised template for the guideline**

The guideline is written according to a standard template developed previously by CERSI-PGx for their guidelines (Dello Russo *et al*, 2026).

**Literature review and prescribing recommendations**

A detailed literature review was previously performed by several members of the writing committee (Murtough *et al*, 2026), which has informed content and recommendations provided in this guideline.

No guidelines exist (to our knowledge) for how to offer *ACKR1*/Duffy-null genotype testing for people taking clozapine. Decisions were made based on best-available evidence and expert input from members of the writing committee. We have consulted with the UK clozapine registries throughout the writing of this guideline, and recommendations were made considering existing UK clozapine prescribing practice. Should UK regulations for clozapine prescribing change in the future, we will update the recommendations in this guideline accordingly.

**Genetic variant for testing**

The rs2814778 c.-67T>C variant in *ACKR1* is the only genetic variant with robust supporting evidence for testing to identify people with ADAN (also known as BEN or DANC). Full details about the rs2814778 variant are described in the main text of the guideline.

**Consultation process**

The final draft of the guideline was shared for consultation with several stakeholders, including UK regulatory agencies, specialist societies, and mental health charities and networks. A full list of bodies we consulted is shown in Supplementary Table 3.

The consultation period was started on 12^th^ February 2026 for one month. All comments received, and our responses, are shown in Supplementary Table 3.

**Patient and public involvement and engagement (PPIE)**

We have collaborated with the Side-By-Side Network, a group of experts by experience in mental health. Bev Chipp (co-author) was the representative member included in the writing committee. The guideline was presented to all members of the Side-By-Side Network in two focus groups on 27^th^ November 2025 and 5^th^ February 2026. The writing committee and members of the Side-By-Side Network co-wrote the plain language summary of this guideline, which is shown below. This summary is also available online via the CERSI-PGx website (<https://cersi-pgx.org/>). The contributions of all lived experience colleagues have been remunerated.

**References for this section**

Dello Russo, C., *et al*. 2026. *CYP2C19* genotype testing for clopidogrel: A guideline developed by the UK Centre of Excellence in Regulatory Science and Innovation in Pharmacogenomics (CERSI-PGx). *Br J Clin Pharmacol*, 92, 329-347. 10.1002/bcp.70370.

Murtough, S., *et al*. 2026. *ACKR1* genetic testing should be offered before starting clozapine treatment. *Nature Mental Health*, 4, 30-41. 10.1038/s44220-025-00554-9.

**PLAIN LANGUAGE SUMMARY**

This summary has been co-written with experts by experience from [the Side-By-Side Network](https://sidebysidenetwork.org/).

**About clozapine**

Clozapine is a medicine that can help people with schizophrenia when other medicines have not worked.

Everyone taking clozapine must have regular blood tests to check their number of white blood cells. This number can drop for some people, which could affect their ability to fight infections. Regular blood tests ensure that a person’s white blood cell count stays above the minimum threshold level. Otherwise, clozapine may have to be stopped.

**About ADAN**

ADAN is a condition caused by a difference in a gene called *ACKR1*. This can affect anyone, but particularly people with African or Middle Eastern ancestry. People with ADAN have naturally low numbers of white blood cells, but they can fight infections normally.

If a person taking clozapine is found to have ADAN, they are monitored on a different scale with a lower minimum threshold level for white blood cells. This usually helps them to continue taking clozapine.

However, ADAN is not always detected. This means that people with undetected ADAN are often unable to take clozapine because their white blood cell level appears too low.

**What is included in the guideline?**

In the guideline, we explain how to test for the genetic difference that causes ADAN in people taking clozapine. It has been designed so that it can be used in the UK’s NHS.

**Who should be offered this pharmacogenetic test?**

The test can be done by taking a single blood sample and only needs to be performed once. The following should be prioritised for the test. People who:

- are about to start taking clozapine.
- have had clozapine stopped in the past because their number of white blood cells became too low.
- are currently taking clozapine and show a low number of white blood cells.

**What should happen when we know the result of the test?**

The test result should be communicated to the person by their doctor.

If the person has the genetic profile for ADAN, the minimum threshold level for white blood cells should be lowered. This will ensure that people with ADAN can safely take clozapine without having it unnecessarily stopped. Following this, the normal monitoring frequency will continue.

If the person does not have the genetic profile for ADAN, they should continue to be monitored on the normal scale for number of white blood cells.

**SUPPLEMENTARY TABLE 1: Guideline committee affiliations, expertise, and conflicts of interest**

| **Name** | **Main Expertise** | **Full Affiliation** | **Institution/s** | **Conflicts of Interest** | **Nation** |
| --- | --- | --- | --- | --- | --- |
| Elvira Bramon | Psychiatry (adults)  **CHAIR** | Professor of Neuroscience and Mental Health  Director [UCL Division of Psychiatry](https://www.ucl.ac.uk/psychiatry/)  Faculty of Brain Sciences  University College London  Hon. Consultant Psychiatrist  North London NHS Foundation Trust | University College London, North London MH Partnership | Member of NHS England Pharmacogenomics Test Evaluation Working Group and NHS England National Genomics Education Programme GeNotes. | England |
| Blanca Bjourson | Psychiatry | Consultant Psychiatrist AMHIC  Clinical Director Acute Mental Health Services (Interim)  Belfast Health and Social Care Trust | Belfast Health and Social Care Trust | None declared | Northern Ireland |
| Vicky Chaplin | Pharmacy  Genomics | Genomics Unit – NHS England | NHS England | No conflicts | England |
| Dharmisha Chauhan | Consultant Pharmacist Genomic Medicine | Consultant Pharmacist Genomic Medicine: | North Thames Genomic Medicine Service Alliance | No conflicts | England |
| Beverley Chipp | Lived experience | Side-By-Side Network | Side-By-Side Network | No conflicts | England |
| Marius Cotic | Genomics | Application Specialist/PhD student  UCL Great Ormond Street Institute of Child Health  Zayed Centre for Research into Rare Disease in Children  20 Guilford Street  London WC1N 1DZ | University College London | No conflicts | England |
| Cinzia Dello Russo | Pharmacology  Pharmacogenomics | Senior Research Associate  Department of Pharmacology and Therapeutics, Institute of Systems Molecular and Integrative Biology (ISMIB), University of Liverpool, Liverpool, UK.  Associate Professor of Pharmacology  Department of Translational Medicine and Surgery, Section of Pharmacology, Università Cattolica del Sacro Cuore - Fondazione Policlinico Universitario A. Gemelli, IRCCS, Rome, Italy | University of Liverpool  Università Cattolica del Sacro Cuore - Fondazione Policlinico Universitario A. Gemelli, IRCCS, Rome, Italy | No conflicts | England |
| Jana De Villiers | Psychiatry | Consultant Psychiatrist - High Secure Intellectual Disability Service for Scotland and N Ireland  Forensic Network Clinical Lead for Intellectual Disability and Autism  The State Hospital, Carstairs, Lanark, ML11 8RP | High Secure Intellectual Disability Service for Scotland and NI | No conflicts | Scotland |
| Olubanké Dzahini | Pharmacy (Mental Health) | Principal Pharmacist  South London & Maudsley NHS Foundation Trust, Denmark Hill, London | Institute of Pharmaceutical Science, King’s College, London | No conflicts | England |
| Frances Elmslie | Clinical genomics | Consultant Clinical Geneticist & Care Group Lead  St George’s University Hospitals NHS Foundation Trust  Clinical Director – South East Genomic Medicine Service Alliance | South East GMSA | None declared | England |
| Katie Evans | Pharmacy (Mental Health) | Lead Pharmacist for Adult Mental Health Cardiff and Vale University Health Board | Cardiff and Vale University Health Board | Vice President of College of Mental Health Pharmacy; Tutor in the Cardiff University Independent Prescribing Course; Assessor in the RPS Advanced Practice Competency Committee; Co-opted representative for CMHP in the RCPsych Psychopharmacology Sub-Committee; NHS Wales Executive in the Adult and Older People Mental Health Clinical Implementation Network; Member of the Pharmacy Delivering a Healthier Wales (PDaHW) Enhancing Patient Experience Working Group  (RPS); member of the HEIW for the foundation pharmacist training development of training packages | Wales |
| Shreyans Gandhi | Haematology | Consultant Haematologist  Kings College Hospital NHS Trust | King's College Hospital NHS Trust | None declared | England |
| Dyfrig Hughes | Health Economics | Co-Director, Centre for Health Economics & Medicines Evaluation,  North Wales Medical School | Bangor University | Chair of NHS Wales National Pharmacogenomics Group, and co-chair of NHS England Pharmacogenomics Test Evaluation Working Group | Wales |
| Huajie Jin | Health economics | Senior Lecturer in Health Economics, Kings College, London | King’s Health Economics  Institute of Psychiatry, Psychology & Neuroscience at Kings College London, London UK | No conflicts | England |
| Daisy Mills | Biochemistry | Research Assistant  Mental Health Neuroscience, Division of Psychiatry  University College London | University College London | No conflicts | England |
| Stephen Murtough | Pharmacy and pharmacogenomics research | Research Fellow  Division of Psychiatry, UCL  4^th^ floor, Maple House  149 Tottenham Court Road  London W1T 7NF | University College London | Member of the GeNotes Mental Health Working Group for the NHS National Genomics Education Programme. | England |
| Daniele Panconesi | Psychiatry | Specialty Registrar – Neuropsychiatry  Department of Neuropsychiatry  National Hospital Neurology and Neurosurgery- Queen Square, London, WC1N 3BG  PhD student  Mental Health Neuroscience  Division of Psychiatry, UCL  4^th^ floor, Maple House  149 Tottenham Court Road  London W1T 7NF | National Hospital Neurology and Neurosurgery  University College, London, UK | None declared | England |
| Munir Pirmohamed | Clinical Pharmacology  Internal Medicine  Pharmacogenomics | Professor Sir  David Weatherall Chair of Medicine and NHS Chair of Pharmacogenetics.  Director, Centre for Drug Safety Science and Wolfson Centre for Personalised Medicine  Director, HDR North  Director, Centre of Excellence in Regulatory Science and Innovation in Pharmacogenomics (CERSI-PGx)  Department of Pharmacology and Therapeutics, Wolfson Centre for Personalised Medicine, University of Liverpool, Liverpool, UK.  Consultant Physician at the Royal Liverpool University Hospital,  Liverpool University Hospital Foundation NHS Trust, Liverpool, UK. | University of Liverpool | Currently receives partnership funding, paid to the University of Liverpool, for the MRC Medicines Development Fellowship Scheme (co-funded by MRC and GSK, AZ, Optum and Hammersmith Medicines Research). He has developed an HLA genotyping panel with MC Diagnostics but does not benefit financially from this. He is part of the IMI Consortium ARDAT ([www.ardat.org](http://www.ardat.org)); none of these of funding sources have been used for the guideline | England |
| Anna Skowronska | Clinical genomics lab scientist | R&D Scientist  West Midlands Genetics Laboratory  Birmingham Women's and Children’s NHS Foundation Trust Mindelsohn Way, Edgbaston  Birmingham, B15 2TG | Birmingham Women's & Children's NHS Foundation Trust | None declared | England |
| Sanjay M Sisodiya | Professor of Neurology | Institute Deputy Director for Sustainability & Climate Change and Consultant Neurologist  Research Department of Epilepsy, UCL Queen Square Institute of Neurology  National Hospital for Neurology and Neurosurgery and Chalfont Centre for Epilepsy  Transformation Director, Epilepsy Society  Lead, ILAE Climate Change Commission and Epilepsy Climate Change | University College London  National Hospital for Neurology and Neurosurgery and Chalfont Centre for Epilepsy | SMS has received honoraria for educational events or advisory boards from Jazz Pharma, Angelini Pharma, Biocodex, Eisai, Zogenix/UCB and institutional contributions for advisory boards, educational events or consultancy work from Eisai, Jazz/GW Pharma, Servier, Stoke Therapeutics, Takeda, UCB and Zogenix. SMS holds the post of Transformation Director at the Epilepsy Society. None of these of funding sources have been used for the guideline. | England |
| Ed Silva | Consultant Forensic Psychiatrist | Specialist Community Forensic Team  for people with Learning Disability and Autism  Hollins Park House  Hollins Park Hospital  Hollins Lane  Winwick  Warrington  WA2 8WA | Specialist Community Forensic Team  for people with Learning Disability and Autism  Hollins Park House  Hollins Park Hospital  Hollins Lane  Winwick  Warrington  WA2 8WA | No conflicts | England |
| Oriella Stellakis | Neuroscience | Neuroscience Mental Health Researcher within the Division of Psychiatry at UCL | University College London | None declared | England |
| Vicky Stinton | Genomics | North West GLH, Manchester University | Genomics Unit, NHS England | None declared | England |
| Sara Stuart-Smith | Haematology | Haematology Consultant  King's College Hospital  Denmark Hill  London  SE5 9RS | King’s College Hospital | No conflicts | England |
| Sarah Tarrant | Diagnostics | Molecular Diagnostics Laboratory Manager NHS Blood and Transplant, Filton, UK | NHS Blood and Transplant, Filton, UK | No conflicts | England |
| David Taylor | Pharmacy  Pathology | Professor of Psychopharmacology,  King’s College London and  South London and Maudsley NHS Foundation Trust | South London and Maudsley NHS Foundation Trust | Funding for research and speaking: Janssen, Otsuka, Lundbeck, Viatris, Rovi, Idorsia  Shareholder in Saladax, Myogenes (a provider of pharmacogenomic tests), 428 Pharma. | England |
| James Walters | Psychiatry (adults) | Deputy Director, Principal Investigator  Professor and director of Cardiff University’s Centre for Neuropsychiatric Genetics and Genomics | Cardiff University | None declared | Wales |
| Michelle Wood | Clinical Scientist | Cardiff and Vale UHB – AWMGS, all-Wales Medical Genomics Service | Cardiff and Vale UHB – AWMGS, all-Wales Medical Genomics Service | No Conflicts | Wales |
| Jessica Woodley | Clinical genomics lab scientist | Clinical Scientist | West Midlands Genomics Laboratory  Central & South Genomic Laboratory Hub  Birmingham Women’s and Children’s NHS Foundation Trust | No Conflicts | England |
| Lauren Varney | Pharmacogenomics | Research Assistant, UCL Division of Psychiatry | University College London | No conflicts | England |

**SUPPLEMENTARY TABLE 2: Differences between the SmPC for clozapine and ZTAS and CPMS guidance**

| **Pre-treatment WBC/ANC thresholds and instructions for starting clozapine** | | | |
| --- | --- | --- | --- |
| SmPC | | ZTAS ^a^ | CPMS ^b^ |
| “Initiation of clozapine treatment must be restricted to those patients with:  WBC ≥ 3.5 × 10^9^/L and  ANC ≥ 2.0 × 10^9^/L  within  standardised normal limits.”  The SmPC does not refer to any traffic-light system. However, this is established UK practice to facilitate communication of results and clinical actions. | | **Green**  WBC ≥ 4.0 × 10^9^/L and  ANC ≥ 2.5 × 10^9^/L  (Treatment may be initiated at discretion of consultant.)  **Intermediate amber**  WBC ≥ 3.5 × 10^9^/L and < 4.0 × 10^9^/L  and/or  ANC ≥ 2.0 × 10^9^/L and < 2.5 × 10^9^/L (Treatment can be initiated by consultant, additional blood monitoring is required.)  **Amber**  WBC ≥3.0 × 10^9^/L and <3.5 × 10^9^/L and/or  ANC ≥ 1.5 × 10^9^/L and < 2. × 10^9^/L (Results indicate patient should not initiate treatment, additional blood sampling required.)  **Red**  WBC < 3.0 × 10^9^/L  and/or ANC <1.5 × 10^9^/L  (Results indicate patient should not initiate treatment and the cause should be investigated.) | **Green**  Required levels for initiation:  WBC ≥ 3.5 × 10^9^/L and  ANC ≥ 2.0 × 10^9^/L  Haematological monitoring is required once the patient starts clozapine treatment. |
| **Monitoring thresholds after clozapine has started ^c^** | | | |
| SmPC | ZTAS | | CPMS |
| “Table 1:  WBC **≥** 3.5 × 10^9^/L and  ANC **≥** 2.0 × 10^9^/L  Action required: continue clozapine.”  No mention of traffic-light system. | **Green**  WBC **≥** 3.5 × 10^9^/L and  ANC **≥** 2.0 × 10^9^/L | | **Green**  WBC **>** 3.5 × 10^9^/L and  ANC **>** 2.0 × 10^9^/L |
| **Increased monitoring frequency instructions for patients returning a low WBC/ANC count ^d^** | | | |
| SmPC | ZTAS | | CPMS |
| No traffic light system mentioned. In the equivalent amber range, the SmPC does not recommend additional clinical observations. It only requires an increased frequency of blood tests to twice weekly.  “If, during Clozapine therapy, either the WBC count falls to between 3.5x10^9^/l and 3.0x10^9^/l or the ANC falls to between 2.0x10^9^/l  and 1.5x10^9^/l, haematological evaluations must be performed at least twice  weekly until the patient’s WBC count and ANC stabilise within the range 3.0-3.5x10^9^/l and 1.5-2.0x10^9^/l, respectively, or higher.” | Amber monitoring:  “A result which is acceptable for continuation of Zaponex treatment but indicates that extra caution must be exercised. The patient’s clinical condition should be closely observed, and additional blood tests are required.”  “In case of amber results, ZTAS advises twice weekly full blood count testing until the counts are back within green  Ranges.” | | Amber monitoring:  No mention of clinical condition.  “If a patient has an amber blood result a full blood count must be performed twice weekly until the count stabilises in this range or increases.” |
| **Criteria for seeking haematology advice/consultation** | | | |
| SmPC | ZTAS | | CPMS |
| Section Low WBC count / ANC:  “If clozapine has been withdrawn and either a further drop in the WBC count below **2.0x10^9^/l** occurs or the ANC falls below **1.0x10^9^/l**, the management of this condition must be guided by an experienced haematologist.” | “If the neutrophil count falls below **0.5x10⁹/L**, or if a patient with neutrophil count below **1.0x10⁹/L** develops a fever, it is strongly advised to contact a haematologist for an appropriate assessment and treatment regimen for the patient.” | | “If the patient’s neutrophil count falls to less than **1.0 × 10^9^/L** or the WBC falls to less than **2.0 × 10^9^/L** OR if the patient develops a fever, it is extremely important to contact a haematologist, or failing this, a general medical physician, for advice regarding appropriate treatment for the patient. This may include transferring the patient to a ward with facilities for the care of neutropenic patients.” |

After reviewing the three clozapine SmPCs and, where available the literature published by the registries, we have identified differences. The differences exist between the clozapine SmPCs and the literature published by the two most widely used clozapine registries, CPMS and ZTAS. We have been unable to access literature for the DMS registry (at the time of writing this guideline) and we have requested this information. We propose that thresholds and monitoring protocols across SmPCs and all three UK registries should be harmonised.

^a^ ZTAS Manual. July 2024.

^b^ Clozaril® Connect. Clozapine, neutropenia and agranulocytosis, red alert management. November 2018.

^c^ The SmPC makes no distinction between the thresholds required for pre-treatment and continuation of clozapine. The symbol for the required minimum WBC/ANC differs between registries.

^d^ Table 1 in the SmPC clearly defines the boundaries of the WBC/ANC thresholds requiring twice weekly blood tests. However, the SmPC text in the section “Low WBC count/ANC” is different to Table 1 for patients with a WBC of exactly 3.5×10^9^/L or ANC of exactly 2.0×10^9^/L. The SmPC does not define what constitutes “stabilised WBC/ANC counts” in the range requiring twice weekly blood monitoring.

**SUPPLEMENTARY TABLE 3: External Consultation Comments and Responses**

| Organisation | Comments received | Responses from the Committee | Changes to the guideline as a result of the consultation (if any) |
| --- | --- | --- | --- |
| **UK Regulatory and/or guideline bodies** | | | |
| MHRA | The MHRA has reviewed the clozapine guideline and provided the following comments.  **ABSTRACT:** The guideline states that ‘It is standard UK practice to lower WBC/ANC monitoring thresholds for people with ADAN, which allows clozapine therapy to start and continue safely’. However, please consider that this is not currently specifically stated on the PI (patient information) of clozapine.  **Section 1.1:** Clozapine is currently contraindicated in “History of clozapine-induced agranulocytosis”. Contraindications in the licence (4.3 of the SmPC) do not say anything about two “red” results on consecutive days  **Section 1.1:** Please note that Denzapine, by Britannia Pharmaceuticals, is also an approved clozapine product in the UK.  **Table 1:** Please note that the information in the product information does not state that it is contraindicated. Instead, it says “Immediately stop clozapine treatment, sample blood daily until haematological abnormality is resolved, monitor for infection. Do not re-expose the patient”. Again, in the bullet points related to the ‘Existing protocol to manage a red alert’ you used the word contraindicated. Please be aware that as mentioned above, clozapine is only contraindicated in patients with “History of clozapine-induced agranulocytosis”.  **Table 1. Note ** DANC/ADAN WBC/ANC threshold:** please note that information on the DANC/ADAN-revised thresholds is not part of the current licence of clozapine.  **Section 1.2 *ACKR1/DARC*-associated neutropenia (ADAN):** in relation to the sentence ‘reduced WBC/ANC monitoring thresholds are applied for clozapine initiation and continuation’, please be advised that the current licence of clozapine does not state reduced initiation thresholds for clozapine. It only says that: “Patients who have low WBC counts because of benign ethnic neutropenia should be given special consideration and may only be started on clozapine with the agreement of a haematologist.”  **Section 4 and Section 7**: Currently the CNRD do not hold data on the readings for a red result. Data will need to be requested to the MAH clozapine monitoring systems to understand whether the red result was within a normal result for an ADAN patient, or if the values would indicate it was a red result also for an ADAN patient  An additional, more general point is that it should be made clearer in section 10.1 that the current SmPCs do not include any information on *ACKR1*/Duffy-null genotype testing. | We thank the MHRA for their helpful comments. Please see our responses below.  We are aware that the SmPC does not include revised thresholds for people with ADAN (also known as benign ethnic neutropenia or BEN). In section 4.4 Special warnings and precautions for use, the SmPC recommends: “*Patients who have low WBC counts because of benign ethnic neutropenia should be given special consideration and may only be started on Clozapine with the agreement of a haematologist”*.  Details reported in this CERSI-PGx guideline reflect routine practice in the UK for clozapine prescribing. This includes the use of revised thresholds for people with confirmed ADAN, which have been agreed with the clozapine providers and monitoring registries and are equivalent across the UK. However, we note that revised thresholds are not reported in the SmPC or the Patient Information Leaflet (PIL) for clozapine. Given that application of revised thresholds for people with confirmed ADAN is routine UK practice, we have not changed the abstract. However, we have revised Section 1.1 of the guideline to state that monitoring is a mandatory requirement provided by the MHRA, although monitoring is regulated and managed by the clozapine monitoring registries. Additionally, we highlight in the final paragraph of this section that revised thresholds for ADAN (as well as the traffic light system used throughout UK practice) are not included in the SmPC for any UK-licensed clozapine containing medicinal products.    We have revised section 1.1, as follows: “If two “red” results are recorded on two consecutive days, clozapine must not be restarted, and the person must be registered with the Central Non-Rechallenge Database.”  We suggest this aligns with text from the SmPC for clozapine: “Confirmation of the haematological values is recommended by performing two blood counts on two consecutive days.”  Additionally, in Table 1, we have replaced “contraindicated” with “clozapine should not be restarted.”  We have amended the text to include Denzapine®. We have also consulted and shared the draft guideline with the three registries including DMS.  We have amended wording in Table 1, stating that clozapine “should not be restarted” (in place of “contraindicated”). In the bullet points section beneath the table, we have rephrased as follows: “In people with a confirmed “red” result, clozapine should not be restarted.”  We are aware of this and have clearly reported this information in section 1.1***.***  We have addressed this point in sections 1.1 and 1.2 with the following two additions:  “…the SmPCs do not mention revised thresholds for people with *ACKR1*/*DARC*-associated neutropenia (described in Section 1.2) or the traffic light system that is used throughout the UK for WBC/ANC monitoring for people taking clozapine.”  “…we note that the revised thresholds are not included in the clozapine product SmPCs but have been agreed by the clozapine providers and are equivalent across the UK.”  As reported in Section 3, multiple studies have shown that ADAN goes frequently undetected among clozapine users, and therefore patients may be erroneously registered in the Central Non-Rechallenge Database (CNRD). In the UK, if a person is registered with the CNRD, any rechallenge with clozapine requires an off-licence agreement with the manufacturer. Both in Section 4 and 7, we wanted to highlight the relevance of providing the pharmacogenetic test for the rs2814778 variant in the *ACKR1* gene to people registered in the CNRD, since we can possibly identify individuals that can indeed benefit from clozapine and in whom the drug can be safely re-started. We also expect that once the pre-emptive test is implemented at full scale, the number of patients with undetected ADAN erroneously registered in the CNRD will progressively be reduced.  This is now clearly described in the guideline. | Yes – Section 1.1  Yes – Section 1.1  Yes – Section 1.1  Yes – Table 1  Yes – Section 1.1  Yes – Sections 1.1 and 1.2  No  Yes – Section 10.1 |
| NICE | No response received. |  |  |
| **UK clozapine registries** | | | |
| Clozaril Patient Monitoring Service (CPMS) | No response received. |  |  |
| Denzapine Monitoring System (DMS) | We thank the members from Britannia Pharmaceuticals and DMS for meeting with us to discuss the guideline, and we note that no formal written response has been provided. |  |  |
| Zaponex Treatment Access System (ZTAS) | Thank you for sending us the documents for our review. Please find our comments in the enclosed document:  **Section 1.1:** The guideline states that: ‘For people with two “red” results on consecutive days, clozapine is contraindicated’. The term “contraindicated” in this context may be slightly misleading. At the point described in the text, clozapine treatment has already been discontinued following a confirmed red result. The clinical implication is that future rechallenge should not occur. For clarity, it may be preferable to rephrase this as: “clozapine should not be restarted” or “future rechallenge with clozapine is contraindicated.”    **Section 1.1:** The guideline states that there are differences between monitoring guidance provided by the clozapine registries. It may be helpful to clarify that the category “intermediate amber” is currently defined only within the Zaponex Treatment Access System (ZTAS) monitoring framework and is not used by other UK clozapine monitoring services or described in the SmPC.  **Section 1.1 (last paragraph):** The text refers to CPMS and ZTAS, but the Denzapine Monitoring System (DMS) is not mentioned.    **Table 1.** The monitoring thresholds presented in Table 1 may change in the future.    **Table 1.** The wording “clozapine is contraindicated” may be misleading in this context. As noted previously, clozapine has already been discontinued following a confirmed red result. It may therefore be clearer to state that clozapine should not be restarted.    **Table 1. Bullet points:** The text states that “the person may resume clozapine treatment.” It may be helpful to clarify that treatment should only be resumed after two green results have been obtained, in line with current monitoring practice. The statement that the person may resume clozapine treatment may require clarification. In current monitoring practice, clozapine should not be restarted until two green results have been obtained. This could be reflected more clearly in the wording.    **Table 1.** Note ** DANC/ADAN WBC/ANC thresholds are provided by the UK’s clozapine registries as part of an off-licence agreement. With respect to this statement, you should consider that where BEN/ADAN has been confirmed by a haematologist, use of the revised monitoring thresholds is considered in-licence within current UK clozapine monitoring practice. The wording should therefore be revised to reflect this distinction accurately.    In this same section, the guideline suggests that confirmation of the Duffy-null genotype alone is sufficient to apply DANC/ADAN monitoring thresholds without further specialist input. However, the SmPC for clozapine products states that patients with low WBC counts due to benign ethnic neutropenia should only be started on clozapine with the agreement of a haematologist. Therefore, even if a genetic test confirms the Duffy-null genotype, involvement of a haematologist remains appropriate to confirm the diagnosis and support clinical decision-making. It may be helpful for the guideline to reflect this requirement to ensure alignment with the SmPC and current monitoring practice. Currently, there is no indication that the MHRA will revise the clozapine SmPC to eliminate the requirement for a Consultant Haematologist to confirm a diagnosis of BEN.    **Table 3:** The headings “All of Us” and “gnomAD” in Table 3 may not be immediately clear to all readers. It may be helpful to briefly clarify that these refer to genomic population databases used to estimate variant frequencies, or to add a short explanatory note in the table legend.    **Section 3. EVIDENCE OVERVIEW.** It may be helpful to clarify that operational processes differ between registries and suppliers, and that rechallenge decisions are case-specific (e.g. chemotherapy induced blood dyscrasia) and subject to regulatory and product information constraints.  **Section 3. EVIDENCE OVERVIEW.** Under current UK product information, application of revised thresholds typically requires haematology confirmation. It may therefore be helpful to reflect this regulatory requirement to ensure alignment with the SmPC and registry processes.    **Section 4. Criterion 2. Testing for people registered in the Central Non-Rechallenge Database.** The wording ‘misdiagnosed with clozapine-induced agranulocytosis’ need clarification. If a patient with BEN/ADAN develops true agranulocytosis (ANC <0.5 × 10⁹/L), registration in the CNRD is appropriate. The text could therefore be rephrased to reflect that only patients incorrectly classified due to BEN-related neutropenia would be candidates for reassessment.      **Section 5. INTEGRATING PHARMACOGENETIC TESTING INTO EXISTING CLINICAL PATHWAYS**. Referring to paragraph 4, it should be noted that our clozapine monitoring system currently does not support SNOMED CT coding. For example, some databases can only record such results as free text entries. It may therefore be helpful to acknowledge that implementation of structured coding (such as SNOMED CT) may require future system adaptations, and that in the interim results may need to be captured in free text fields within existing monitoring databases.  **Section 5. INTEGRATING PHARMACOGENETIC TESTING INTO EXISTING CLINICAL PATHWAYS.** Referring to the last paragraph, the text correctly notes that individuals with the Duffy-null genotype may have ANC values within the normal range. Conversely, not all individuals with the Duffy-null genotype necessarily have benign ethnic neutropenia (BEN).  **Section 7.1.** The CERSI-PGx guideline states that ‘Some UK clozapine registries require haematology authorisation to apply the DANC/ADAN-revised thresholds. We recommend that a test result confirming the Duffy-null genotype is sufficient to apply DANC/ADAN-revised thresholds without delay’. However, the current SmPC for clozapine states that patients with low WBC counts due to benign ethnic neutropenia should only be started on clozapine with the agreement of a haematologist.  Therefore, removal of haematology authorisation would only be possible if the MHRA were to revise the SmPC wording accordingly. Until such a regulatory change occurs, application of revised thresholds without haematology involvement would not be consistent with the current product information and monitoring framework.    **Section 7.1.** The CERSI-PGx guideline states that ‘If clozapine rechallenge is considered clinically appropriate, removal of the person from the Central Non-Rechallenge Database should be requested’. However, in current practice removal from the CNRD is generally not possible. Instead, a note can be added indicating that the patient has been rechallenged. Indeed, currently it is not possible to remove patients from the CNRD as per MHRA decision but they can be reactivated (with the reason for re-activation provided e.g. no longer red criteria, clinical decision)  **Figure 1.** The reference to abnormal growth and development may not be relevant in this context, as clozapine is licensed in the UK for patients aged 16 years and older. It may therefore be appropriate to reconsider whether this point is necessary in the guideline.    **Figure 1.** The involvement of a haematologist is currently mandated via the SmPC, which has been approved by the MHRA. It may therefore be helpful for the guideline to acknowledge this requirement to ensure alignment with the existing regulatory framework.    **Section 8.2:** The statement that norclozapine is an “active but clinically irrelevant metabolite” may be too definitive. Norclozapine has pharmacological activity and may contribute to the overall clinical effects of clozapine. It may therefore be preferable to avoid characterising it as clinically irrelevant and instead state that its clinical contribution is considered limited. Clinical data from clozapine-treated patients suggest a correlation between norclozapine serum levels and metabolic syndrome parameters like waist circumference and HbA1c. Norclozapine also contributes to sedation.  **Table 4. Criterion 3.** The monitoring thresholds may change in the future.    **Section 10.1 Summary of Product Characteristics (SmPC) and the UK Monitoring System**. The monitoring may change in the future.  **Section 10.** The guideline states ‘However, we note there is no relationship between risk of cardiac adverse events with clozapine and ACKR1’ This comment is not completely clear. We found a relevant reference: The Duffy-null allele is a common mutation in the ACKR1 gene, especially prevalent in individuals of African ancestry, which results in the loss of ACKR1 expression on red blood cells. This erythroid-silent ACKR1 deficiency is linked to increased inflammation and a higher burden of CVD in African Americans.  Guha A, Wang X, Harris RA, Nelson AG, Stepp D, Klaassen Z, Raval P, Cortes J, Coughlin SS, Bogdanov VY, Moore JX, Desai N, Miller DD, Lu XY, Kim HW, Weintraub NL. Obesity and the Bidirectional Risk of Cancer and Cardiovascular Diseases in African Americans: Disparity vs. Ancestry. Front Cardiovasc Med. 2021 Oct 18;8:761488. doi: 10.3389/fcvm.2021.761488. | We thank the members from ZTAS for their insightful comments and for taking the time to meet with the authors and to review this guideline.  We have amended the text according to your suggestion (as well as the feedback from the MHRA), as follows: “If two “red” results are recorded on two consecutive days, clozapine must not be restarted, and the person must be registered with the Central Non-Rechallenge Database.”      We have highlighted differences between the clozapine registry literature in Supplementary Table 2, including the “intermediate amber” category.  This paragraph has been revised to mention all three registries more clearly. The main message is that none of the SmPCs provide revised thresholds for ADAN/DARC/BEN and that only the literature from the registries does. At the time of writing this guideline, we were unable to access any DMS literature beyond the SmPC for Denzapine. We have requested this literature from the DMS registry. Based on the information available, we report detailed differences between the SmPC and available literature from registries in Supplementary Table 2. The guideline proposes that harmonisation across the SmPCs and all literature from the three registries is desirable.    We have addressed this in section 10.4, highlighting that changes in clozapine monitoring requirements have occurred at international levels (in the US and across the EU) and that the guideline will be promptly revised should changes occur to the regulatory framework in the UK. The text is as follows: “In the instance that clozapine monitoring guidelines change in the UK, this guideline will be adapted to map to new monitoring thresholds and protocols. As with recommendations in the USA and Europe, monitoring thresholds for ADAN should be revised so that they are lower than any standard thresholds.”    We have amended the text as per this suggestion.        We have amended the text as per this suggestion.            We have corrected this statement about the off-license agreement. Here, we were specifically highlighting the fact that revised thresholds for the monitoring of people with ADAN are not listed in the SmPC of any clozapine containing medicinal products that are licenced in the UK. Rather, the SmPC states that “Patients who have low WBC counts because of benign ethnic neutropenia should be given special consideration and may only be started on clozapine with the agreement of a haematologist.”    We are aware that our recommendations on this point differ from existing UK practice and we have made this clearer in Table 1. We believe it is essential to suggest a change where, based on the genetic test result, all people with the Duffy-null genotype should be monitored using revised WBC and ANC thresholds without the need for haematology confirmation. The prescriber should action this promptly. This is to ensure that correct thresholds for these people are applied without delay. Evidence from the literature supports our recommendation, as people with the Duffy-null genotype are more likely to have lower ANCs (relative to ‘normal values’) and are at risk of clozapine exclusion and discontinuation. Our recommendation has been informed by haematologists from our writing committee. To ensure safety, we also provide guidance in Section 7.2 and Figure 1 about when haematology advice should be sought. Briefly, this includes patients who record a WBC <2.0 × 10^9^/L and/or ANC <1.0 × 10^9^/L alongside a relevant clinical feature, such as sustained temperature ≥38ºC (please see Figure 1 for a full list of relevant clinical features). In addition, we have included a note in Figure 1 that haematologist approval may be needed (at the time of writing and according to existing UK practice) to align with existing requirements for clozapine registries to apply revised thresholds.    We have clarified in the legend that data reported in the table refer to genomic studies and population databases used to provide estimates of variant frequencies.      We have revised the text to include these suggestions.        As stated above, we are aware that our recommendations differ from existing UK practice, however we suggest that people with the Duffy-null genotype should be monitored using revised WBC and ANC thresholds without the need for haematology confirmation. These has been agreed with haematologists and is supported by robust evidence.    We have amended the text accordingly: “after being incorrectly classified with clozapine-induced agranulocytosis due to undetected ADAN”. We prefer to use ADAN in place of BEN.        We have included that future system adaptation may be required to use SNOMED CT coding. We have included the following text: “However, we are aware this may require adaptations to existing registry systems, and we recommend that delays to system changes should not hinder the use of the test or application of revised thresholds. In the immediate future, test results may be recorded according to existing system requirements (such as being captured in free text fields), to ensure quick application of revised thresholds for people with the Duffy-null genotype.”    Please note, we have explained in this section that people with the Duffy-null genotype are significantly more likely to have a lower ANC as well as transient neutropenia (making them at risk of developing benign neutropenia).      As stated above, we are aware that the SmPC mandates that “Patients who have low WBC counts because of benign ethnic neutropenia should be given special consideration and may only be started on Clozapine with the agreement of a haematologist”. However, we know there is robust evidence to safely start clozapine in these people by adopting revised thresholds. Our recommendation has been developed and agreed with haematologist members of our writing committee. In addition, CERSI-PGx is working with the MHRA to streamline the regulatory pathways for pharmacogenetic implementation in routine practice in the NHS. The MHRA is therefore aware of the content of our guideline and has provided feedback as reported above.  Moreover, we have noted in Figure 1 that clozapine registries may require haematologist approval to apply revised thresholds – which aligns with existing UK practice.    We thank the ZTAS team for highlighting this important point. We have revised our text to align with these recommendations: “For people registered with the Central Non-Rechallenge Database (i.e. tested under criterion 2), clozapine rechallenge under DANC/ADAN-revised thresholds should be considered by the managing prescriber and clinical team. At present, it is not possible to remove a person from the Central Non-Rechallenge Database, however a note may be added to the database system stating that the person has been rechallenged.”      By including this clinical feature, haematologists will be able to identify (or rule out) people with rare forms of neutropenia that are known to affect growth and development. Some examples include Shwachman-Diamond syndrome, cartilage hair hypoplasia, and Barth syndrome. Therefore, it is important to assess people presenting with neutropenia who also have abnormal growth and development.    We agree it is useful to highlight that our recommendations differ from existing UK practice. We also note that our recommendations have been made with input from haematologist members of our writing committee.  We have included text in Figure 1, stating that “Some clozapine registries currently require haematologist approval to apply revised thresholds for DANC/ADAN (also known as BEN).”    We have amended this section.              As mentioned in Section 10.4, we will update the guideline following any changes in the regulatory framework that may occur in the future.    As above, we will amend the guideline according to any changes in the regulatory framework that may occur in the future.    To our knowledge, there is no robust evidence demonstrating that ACKR1 variants are associated with cardiac adverse events due to clozapine. We acknowledge the cited reference; however we have also found evidence suggesting *ACKR1* may have a protective role in the context of atherosclerosis (Wan W et al. Atypical chemokine receptor 1 deficiency reduces atherogenesis in ApoE-knockout mice. Cardiovasc Res. 2015 Jun 1;106(3):478-87. doi: 10.1093/cvr/cvv124). Furthermore, there are no data linking this variant to clozapine-induced myocarditis. Therefore, taken collectively the evidence does not suggest a link between *ACKR1* and cardiac ADRs due to clozapine. We have therefore left the text as originally written. | Yes – Section 1.1  No  Yes – Section 1.1 and Supplementary Table 2  Yes – Section 10.4  Yes – Table 1  Yes – Table 1  Yes – Table 1  Yes – Table 1  Yes – Table 3  Yes – Section 3  No  Yes – Section 4  Yes – Section 5  No  No  Yes – Section 7  No  Yes – Figure 1  Yes – Section 8.2  No  No  No |
| **Professional colleges and/or societies** | | | |
| College of Mental Health Pharmacists | In general, we feel the guidelines are well written.  In some sections, there is perhaps unnecessary detail, for example in section 7, when describing "actions if genotype detected", it would be preferable to simply say " Follow DANC revised thresholds", rather than outlining what to do for GREEN, AMBER and RED.   Similarly, with "actions if genotype not detected", say "Follow standard protocol".    In section 11, the guidelines mention serological testing for a person’s Duffy blood group.  The guidance doesn't include any information about this serological testing, such as the cost, validity or reliability of these antigen tests.    Without this, we feel this should not be recommended.  In previous sections, the guidance states that if genetic testing is not available, patients should follow the standard clozapine protocol.  We feel that this should be repeated here i.e. if a person cannot have a genetic test, for whatever reason, then they should follow the standard clozapine protocol.    Finally, we think the guidance should acknowledge the known regional variation in ethnicity across the UK, as this would have implications for the health economics. Currently the health economic data is based on an assumption that 3.85% of the UK population have the Duffy-null genotype.  However, we know that this genotype is not spread evenly across the country.  In a region that has over 90% European descendants, the incidence of this genotype would be much lower.  And in the more ethnically diverse regions, it will be much higher.   If we had a national agreement to pay for the test, then this would not matter.  However it is more likely that for example in England, individual ICBs are going to consider adopting this test within their region, similar to how they have done with clopidogrel. Therefore, it would be helpful to add a comment highlighting that if national adoption is not approved, local leaders should consider its applicability to their population, bearing in mind that patients with African or Middle Eastern heritage are more likely to benefit from this test.  We acknowledge the difficulty in sometimes getting a diagnosis of BEN from haematology even with the patient having the required ethnicity so can see the value in genetic testing. | We thank the College for their thoughtful and helpful comments. We have responded to each comment one-by-one below.  We agree the text should be written simply and without excess information. We have removed this text in line with the College’s suggestions.  Serological testing for Duffy antigen expression is a common assay and should be routinely available from most hospital transfusion laboratories. Please consider the comments by NHS Blood and Transplant. However, we agree that genotype testing for the rs2814778 c.-67T>C variant in *ACKR1* is the preferred method for identifying ADAN, not least because it is this variant specifically that has been associated with ADAN and benign neutropenia. We have amended this section, making clear that serological testing should only be used when genotype testing is unavailable. In these instances, and in the absence of genotype testing, it may be beneficial for the patient to be tested using this method. To ensure that a correct diagnosis of ADAN is made, we have added an additional recommendation that a haematologist should provide input to interpret a person’s neutrophil count in light of a serological test result. In future, if other variants in *ACKR1* (that also cause a Duffy-null phenotype) are associated with benign neutropenia, the validity of serological testing may become more justified.  We agree that rates of people with the Duffy-null genotype will differ substantially across the UK. Because *ACKR1* genetic testing is not routinely available, for health economics we had to estimate the overall prevalence figure of 3.85% Duffy-null from the ancestry groups listed in the 2021 censuses covering all four UK nations. However, we want to highlight that self-reported ethnicity is an unreliable measure of genetic ancestry. The *ACKR1* genetic test should be offered to everyone regardless of their ancestry. To support this, we have added text to Section 11 (Other Considerations) as follows:  “We previously estimated the overall prevalence rate of the Duffy-null genotype in the UK to be 3.85%.^36^ In the absence of real-world genetic testing data, we calculated approximate overall prevalence using ethnicity data self-reported in the 2021 Censuses of the four UK nations alongside Duffy-null variant frequencies. In reality, Duffy-null prevalence will vary across the UK depending on the genetic ancestry of a given local population. For instance, in areas with a higher proportion of people with African or Middle Eastern genetic ancestry, there should be more people with the Duffy-null genotype. If the *ACKR1* genotyping test is not nationally adopted in the UK but is instead offered locally by individual NHS Integrated Care Boards, local leaders may attempt to determine the need for *ACKR1* testing by considering the ethnicity make-up of the local population. We stress that self-reported ethnicity is not an accurate or suitable way to decide who should be offered *ACKR1* genetic testing.^50,51^ Indeed, evidence shows that people identifying as “White” (who may be assumed to have European genetic ancestry) can have the Duffy-null genotype.^23,40^ Due to this, we advise that genetic testing should be offered irrespective of the ethnicity make-up of a given local population, even if the test is considered for approval on a local basis.” | Yes – Section 7 (Clinical Actions Based on Genotype)  Yes – Section 11 (Other Considerations)  Yes – Section 11 (Other Considerations) |
| UKCPA genomics committee | We found this to be a very informative and clear guideline. The explanation of monitoring practices for the UK is very clear and well presented.  This has more of a diagnostic / stratification utility than PGx test and this should be explained and linked to lack of guidelines from international PGx consortiums. A general point, while we think it is essential that CERSI-PGx publish this guideline to improve access to clozapine, reduce inequalities, and support safe and effective prescribing. If, by some definitions, a pharmacogenetic test determines a response to medication then this is not a pharmacogenetic test. Rather it is a diagnostic test for ADAN/DARC which does not affect a person’s response to clozapine but rather determines how they are monitored when taking clozapine considering this diagnosis. This may be why other pharmacogenetics guidelines and databases have not included this drug-gene pair. Some words in the introduction or section 8 or section 11 may be useful to explain this.  The interchanging of the terms “Duffy-null variant” and “Duffy-null genotype” can be confusing in Section 1.2. “Duffy-null variant” is not included in Table 2. In the footnote to Table 2 it is explained that “’Duffy-null genotype’” is used throughout the guideline to refer to people who are homozygous for the T>C variant”. However later in the same section and in Table 3 the term “Duffy-null variant” is used. For example, in Section 2.1 states that the Duffy-null genotype prevalence in African ancestries is approximately 80%. Then in Table 3 the frequency of the Duffy-null variant is given as 84%.  It may help to include the genotype (C/C, C/T, or T/T) throughout the document each time genotype is referred to for further clarity. For example, “Duffy-null genotype” in the document would always become “Duffy-null genotype (C/C)” etc  It would be helpful to include an explanation that the term “Duffy antigen” is a synonym for the *ACKR1* protein, as I couldn’t see this included anywhere. For example: “*ACKR1* is also known as Duffy antigen chemokine receptor (DARC)”. And “*ACKR1* protein is also known as Duffy antigen”. This would explain the terminology better and could be included in the text, or in Table 2.  The phenotype terms DANC, ADAN, and BEN are depicted as interchangeable terms, with BEN being the historic term, but BEN may relate to a wider phenotype of genetic causes, while an ACKR1 variant may have more precise scope. Equally, it would be good to explore the evidence of other genes causing BEN e.g. CXCL2, CDK6, CSF3, and their relevance here or mentioned in the research section, if the evidence demonstrates a signal that is relevant to clozapine monitoring e.g. https://pmc.ncbi.nlm.nih.gov/articles/PMC6702066/  Include the ® symbol where proprietary drug names are used.  **SNOMED CT Codes**  There are some other potentially relevant codes in the SNOMED CT Browser:  Benign ethnic neutropenia (disorder) SCTID: 1156300000  Duffy antigen type (finding) SCTID: 250390009  However, agree that new codes are needed to update to approved terminology and to include recording of a negative result so that it is clear in the notes that a patient has been tested to avoid unnecessary repeat testing. Has the CERSI-PGx group requested these new codes from the data and digital team at NHSE? As it can take up to 12 months to complete approval for new codes.  Generalised content: Given the wealth of general clozapine information available via the risk minimisation programmes, I felt it was perhaps not necessary to repeat certain parts of this information in this guideline: Pg 19 – cardiotoxicity singled out as a clozapine AE. Is it necessary to state non ACKR1 related AEs? If so, there are several other potentially serious AE in addition to cardiotoxicity  Section 7.2 - for patients without ACKR1 variants, beyond advice for informing patients of results, NOT rechallenging and recording ACKR1 results in medical records, is it necessary to go on to state the standard guidelines for the management of clozapine patients – could a reference be made to Table 1?  Neutropenic sepsis section; Which guideline are the defining parameters for neutropenic sepsis based on? This should be referenced, as currently the NICE CG is referenced, but temperature and neutrophil parameters stated, differ from main NICE definition.  Figure 1; This is a great flow chart. Although informing the patient of their result is mentioned on the previous page, it would be great to also include this on the flow chart? In the neutropenic sepsis information box, should ‘neutropenia’ be defined?  Working Group representation/stakeholder engagement: Have clozapine manufacturers/those overseeing the Central Non-Rechallenge Database been involved in the guideline development? Are all relevant parties in agreement with recommendations to rechallenge patients on the database subsequently diagnosed with ADAN? Is it worth stating which programmes require confirmation of diagnosis by a haematologist?  Governance: If a patient presents with abnormally low WCC/ANC at baseline and ACKR1 variant is not detected, this requires further investigation by a haematologist- should this be mentioned?  Section 7.1: when rechallenging positive patients, who else in the MDT should be involved? Is there any data on success rates if patients are re-prescribed clozapine in this cohort?  Regulatory considerations; Is the MHRA clozapine review still ongoing? If so, perhaps it could be mentioned in section 10, as information on the review is in the public domain? MHRA to review blood monitoring requirements for clozapine patients - The Pharmaceutical Journal  Homozygous rs2814778 is a strong genetic marker associated with ADAN/Duffy-null associated low neutrophil counts, but genotype should be interpreted alongside serial counts and clinical assessment. Further discussion on genotype to phenotype associations and clinical impact may be beneficial, also including information on penetrance and heterogeneity.  Research recommendations section: Consider the penetrance and heterogeneity of ACKR1 rs2814778 C/C and its interactions with other background diseases, for example, sickle cell disease has an epistatic interaction where ADAN can have a smaller effect size, and SCD is often prevalent in similar ethnic groups.  tps://haematologica.org/article/view/10828  QR code - QR codes alone may have limited utility. Consider adding both the actual hyperlink as the guideline will more likely be read on a computer screen, and the website’s title and revision date in case the link changes in the future so it can be searched. Using a QR code without a hyperlink means you need to switch to a smaller device and assumes a smartphone is available. | We thank the UKCPA Genomics Committee for their detailed and helpful comments. We have responded to each comment below.  We believe *ACRK1* genotyping for people taking clozapine constitutes a pharmacogenetic test.  This test facilitates safe access to a medicine and has a direct impact on its prescribing. We agree that a narrower definition of what constitutes a pharmacogenetic test – such as testing for gene variants that affect drug response – could explain why guidelines up till now have not been developed for *ACKR1* and clozapine.  While it is beyond the scope of this guideline to define what constitutes a pharmacogenetic test, we favour a broader definition, and we thank UKCPA for highlighting this interesting point.  We agree that it would be useful to provide clarity for the reader here. In Table 2, we have added the following text: “Duffy-null variant is used when referring to the T>C variant, rather than the combination of alleles at rs2814778 (i.e. a person’s genotype).”  We have also added additional text to the legend of Table 3. There is variation in frequency of the Duffy-null variant *and* Duffy-null genotype (homozygous carriers of the T>C variant), even among people with ancestors from similar geographical regions. This is demonstrated by an analysis by Howes *et al* (2011) which showed that frequency of the Duffy-null genotype in some parts of Sub-Saharan Africa (such as The Gambia) is predicted to reach fixation (i.e. 100%), while being lower in other parts of the continent. Figures from the two datasets (All of Us and gnomAD) shown in Table 3 refer to frequency rates of the Duffy-null variant, rather than the Duffy-null genotype, as these data are provided by both data browsers (while percentage values of homozygotes are not provided). To ensure clarity for the reader, we have added new text to explain the data included in Table 3.  We agree that the text should be clear to the reader. We have listed the relevant genotype as per your suggestion in the section “Clinical actions based on genotype” as it is key for the interpretation of the test result. As “Duffy-null genotype” always refers to the C/C genotype, we suggest that in the rest of the text it is not necessary to include the base pairings after each mention of the term, “Duffy-null genotype”. We also refer the reader to Table 2, where terms are described in detail.  We agree with this comment and have added text to clarify the meaning of “Duffy antigen” in all instances where the term is mentioned in the text. For instance, in Table 2, we have added the following: “Duffy antigens (protein products of the *ACKR1* gene)”.  We thank UKCPA for highlighting this interesting point. To date, evidence for variants in other genes that are associated with benign neutropenia are limited. We agree that some evidence – at the GWAS level – suggests variants in other genes may be involved. However, more research is needed to test whether these variants are robustly associated with benign neutropenia not linked to increased risk of infection. If robust evidence demonstrates the involvement of other genes, then a new encompassing term should be considered. At the time of writing, only the rs2814778 homozygous variant in *ACKR1* (i.e. the Duffy-null genotype) has been strongly associated with benign neutropenia, including in cohorts of people taking clozapine. In the section “research recommendations” we have indicated the need to study HLA variants which could be influencing neutropenia/agranulocytosis in clozapine users.  We thank UKCPA for spotting this – we have amended the text to include the ® symbol.  We agree that new terms specifically relating to *ACKR1* genotype would be most appropriate for recording in electronic health records, and we will request these from the relevant team at NHSE. We thank UKCPA for highlighting the expected timeframe for approval.  We thank UKCPA for this comment. The risk of myocarditis or cardiomyopathy is also highlighted (‘singled out’) in the SmPC, together with potential risk of agranulocytosis in a warning box at the beginning of the SmPC for all clozapine containing medicinal products licensed in the UK. Therefore, we have reported this warning verbatim in the guideline.  We agree and have removed this text to simplify the recommendations. In place of the text, we refer to directions given in Table 1, as recommended.  We have amended the text to include the definition of neutropenic sepsis, as provided by NICE: <https://cks.nice.org.uk/topics/neutropenic-sepsis/>. We have also included the NICE definition for neutropenic sepsis in the flow diagram in Figure 1.  We agree and have made the suggested changes to the flowchart. This includes the NICE definition for neutropenic sepsis.  We have consulted with the manufacturers of Clozaril®, Denzapine®, and Zaponex®, who oversee the three clozapine registries operating in the UK. We are aware that our recommendations differ from existing UK practice, however we suggest that people with the Duffy-null genotype should be monitored using revised WBC and ANC thresholds without the need for haematology confirmation. Evidence from the literature supports our recommendation, as people with the Duffy-null genotype are more likely to have lower ANCs (relative to ‘normal values’) and are at risk of clozapine exclusion and discontinuation. Our recommendation has also been informed by haematologists from our writing committee. To ensure safety, we provide guidance in Section 7.2 and Figure 1 about when haematology advice should be sought. Briefly, this includes patients who record a WBC <2.0×10^9^/L and/or ANC <1.0×10^9^/L alongside a relevant clinical feature, such as sustained temperature ≥38ºC (please see Figure 1 for a full list of relevant clinical features). In addition, we have included a note in Figure 1 that haematologist approval may be needed (at the time of writing) for clozapine registries to apply revised thresholds.  We have included guidance in Section 7 and Figure 1, stating that people with a WBC <2.0×10^9^/L and/or ANC <1.0×10^9^/L alongside a relevant clinical feature (please see Figure 1 for a full list of clinical features) should be managed with input from haematology. This guidance applies to all people, irrespective of *ACKR1* genotype, and has been informed by existing guidance in the clozapine SmPC and with expert input from haematologists from our writing committee based on relevant neutropenia guidelines.  In section 7, we have included new text, stating that clozapine rechallenge should be undertaken by the managing prescriber and clinical team. Data from Prof. David Taylor’s studies suggest that rechallenge can be successfully performed in people with undetected ADAN registered in the CNRD (Oloyede E et al. *Schizophr Bull*. 2021. doi: 10.1093/schbul/sbab006).  Details reported in this CERSI-PGx guideline reflect routine practice in the UK, at the time of writing, and are based on the current regulatory framework. We have clarified that if changes to the SmPC are made by the MHRA, including different monitoring requirements, as reported in *The Pharmaceutical Journal* (<https://pharmaceutical-journal.com/article/news/mhra-to-review-blood-monitoring-requirements-for-clozapine-patients>), we will update the guideline accordingly. Please note that we have mentioned this possible development in Section 10.4: ‘If clozapine monitoring guidelines change in the UK, this guideline will be adapted to map to new monitoring thresholds and protocols’.  The guideline advocates monitoring Duffy-null cases (using the revised blood monitoring threshold) well in line with your suggestion of interpretation alongside serial counts. To ensure safety, we have included guidance (in section 7 and Figure 1) about when haematology guidance is required. Evidence shows that all people with the homozygous variant at rs2814778 (Duffy-null genotype) are significantly more likely to have an ANC below the “normal” range (for people with European ancestry) than people carrying a copy of the functional allele. This has been demonstrated with analyses of several large datasets, including the UK Biobank. Given these data, we recommend that all people with the Duffy-null genotype should be monitored with revised monitoring thresholds for ADAN/DANC. We acknowledge that some people with the Duffy-null genotype may present (at least, initially) with an ANC within the “normal” range, however evidence shows that these people are at risk of developing transient neutropenia, making them at risk of disruption or discontinuation of their clozapine treatment. Please see section 1.2 for more detailed discussion about this.  We acknowledge that some people with the Duffy-null genotype may present with an ANC in the “normal” range. However, evidence shows that people with this genotype are at risk of transient neutropenia. One clinical trial found that 33% of Duffy-null individuals (65/199) presented with at least one ANC result <1.5 × 10^9^/L over a six-month period, (Kelly et al, 2024, DOI: 10.1016/j.schres.2023.08.002). We also acknowledge the evidence cited by the UKCPA regarding a potential epistatic interaction between sickle cell disease and ADAN, however we suggest further evidence is needed to influence clinical recommendations.  We thank UKCPA for highlighting this. We agree and have added the hyperlink to Figure 1, along with the NICE definition for neutropenic sepsis. | No  Yes – Table 2 and Table 3 in Section 1.2  No  Yes – Table 2  No  Yes – throughout the text  No  No  Yes – Section 7  Yes – Sections 7.1 and 7.2, and Figure 1  Yes – flowchart (Figure 1)  No  No  Yes – Section 7.1  Yes – Section 10.4  No  No  Yes – Figure 1 |
| Royal College of General Practitioners | We have summarised responses below from members of the Royal College.   1. Based on this information, I would be supportive that this testing should take place, and it is important that patients that have been tested have a correct SNOEMD code to reflect this which is clear to all across secondary / primary care interface. It is important that the wording on the result is understandable to patients and clinicians in a ‘just in time’ learning terminology (probably using a bioinformatician, GP, consultant, and patient in this process and ideally it will be piloted). 2. If genotyping is brought in, then it should be entirely handled by psychiatry, including the pre-test counselling, phlebotomy, and post-test counselling / management. | We thank the Royal College of General Practitioners for their support and comments.  We agree that a relevant and understandable SNOMED code will be useful to record the genetic test result. We hope to request these codes imminently, and we will consider input from members of our writing committee as well as members of a lived experience PPIE group.  We agree that the responsibility for ordering, managing, and interpreting the genetic test should sit with the clozapine prescriber, which in most cases is within psychiatry. Our intention is that the genetic test will not substantially alter the existing clinical pathway for clozapine prescribing. However, we envisage a small number of cases where clozapine may be prescribed in neurology settings (for people with psychosis in Parkinson’s disease) and primary care (although we agree that these are rare occurrences). In these situations, we suggest that the genetic test should be made available to the patient’s prescriber. | No  No |
| Royal Pharmaceutical Society | No response received. |  |  |
| Royal College of Psychiatrists | No response received. |  |  |
| British Society for Haematology | No response received. |  |  |
| National Pharmacogenomics Group (NPGG) NHS Wales | No response received. |  |  |
| NHS Wales Mental Health Network | No response received. |  |  |
| NHS Blood and Transplant | Overall I think this will be very helpful, some formal guidance on this is overdue.  The approach taken should be safe if conservative.  I appreciate the guidance is intended as a framework for genetic testing but is it worth commenting that serological typing for Fy(a) and Fy(b) would also be valid to support the diagnosis of BEN? This is likely to be available with a shorter turnaround time as most hospital transfusion labs will be able to offer this. It may also be worth pointing out there are other described mechanisms of DARC silencing which would be detectable serologically but not with genotyping for the -67T>C variant. | We thank NHS Blood and Transplant for their helpful comments.  We have updated our recommendation in Section 11 (Other Considerations).  The text now states that if genotype testing is unavailable, serological testing for expression of Duffy antigens, Fy(a) and Fy(b), may be considered as an alternative. However, please note we have provided a cautionary statement that only the rs2814778 c.-67T>C variant in *ACKR1* has been robustly associated with ADAN and benign neutropenia. Therefore, serological testing may identify people with other variants in *ACKR1* that also cause a Duffy-null phenotype. Further research is needed to determine whether other variants in *ACKR1* are associated with benign neutropenia. | Yes – Section 11 |
| ABPI | No response received. |  |  |
| BIVDA | No response received. |  |  |
| **Pharmacogenomics groups** | | | |
| ClinPGx | No response received. |  |  |
| DPWG | No response received. |  |  |
| ACGS | No response received. |  |  |
| UK-IPN | Having reviewed the guideline, it is very thorough and well written, and UK-IPN does not have any additional comments to add. | We thank UK-IPN for their supportive comments. | No |
| **Patient organisations and lived experience charities** | | | |
| Clozapine Support Group UK | No response received. |  |  |
| MQ | No response received. |  |  |
| McPin Foundation | No response received. |  |  |
| Rethink Mental Illness | My colleagues are unable to respond however they found your email interesting. | We thank Rethink Mental Illness for their interest in the guideline, and we welcome any further comments in the future. | No |
